# Diabetes exacerbated sepsis-induced intestinal injury by promoting M1 macrophage polarization *via* miR-3061/Snail1 signaling

**DOI:** 10.3389/fimmu.2022.922614

**Published:** 2022-09-09

**Authors:** Fang Tan, Yuling Cao, Lei Zheng, Tao Wang, Shuhua Zhao, Jiong Chen, Changji Pang, Weiyi Xia, Zhengyuan Xia, Ningning Li, Xinjin Chi

**Affiliations:** ^1^ Department of Anaesthesiology, the Seventh Affiliated Hospital of Sun Yat-Sen University (SYSU), Shenzhen, China; ^2^ Tomas Lindahl Nobel Laureate Laboratory, The Seventh Affiliated Hospital of Sun Yat-Sen University (SYSU), Shenzhen, China; ^3^ Department of Anaesthesiology, the First Affiliated Hospital of Guangzhou Medical University, Guangzhou, China; ^4^ State Key Laboratory of Pharmaceutical Biotechnology, Department of Medicine, The University of Hong Kong, Hong Kong SAR, China; ^5^ The University of Hong Kong, Hong Kong, Hong Kong SAR, China

**Keywords:** sepsis, diabetes, intestinal injury, macrophage polarization, miR-3061, Snail1

## Abstract

**Background:**

Macrophages play important roles in diabetes and sepsis-related intestinal injury. Accumulating evidence suggests that microRNAs (miRNAs) act as the fundamental link between macrophage polarization and tissue injury. However, the underlying mechanisms of miRNAs in regulating macrophage polarization–related intestinal injury under diabetes and sepsis conditions remain unclear.

**Methods:**

The cecal ligation and puncture (CLP)–induced sepsis models were established in male wild-type (WT) and diabetic mice. Clodronate liposome was used to deplete macrophage. H&E staining, inflammatory cytokines [tumor necrosis factor–α (TNF-α), interleukin-1β (IL-1β), and IL-6], and intestinal mucosal barrier function markers [occludin, ZO-1, lipopolysaccharide (LPS), and intestinal fatty acid binding protein (iFABP)] were used to assess elevated intestinal damage. miRNA array, RNA-seq, and bioinformatic analysis were performed to detect the miRNA and messenger RNA (mRNA) expression and the potential regulation mechanism. *In vitro*, RAW264.7 cells were cultured in the absence or presence of high glucose and LPS, miR-3061 mimics, and Snail small interfering RNA stimulation, respectively, for further mechanism studies. Luciferase reporter assay was used to confirm the interplay between miRNA and its target genes.

**Results:**

Compared with WT CLP mice, the diabetic CLP mice showed severe intestinal damage characterized by significant increases in Chui’s scores, expression of inflammatory cytokines (TNF-α, IL-1β, and IL-6), serum LPS and iFABP concentration, and significant reductions in tight junction protein occludin and ZO-1 levels. Macrophage depletion reversed the intestinal damage caused by CLP. The bioinformatic analysis revealed that miR-3061/Snail1 might be a potential regulation axis of macrophage polarization. Furthermore, high glucose and LPS stimulation increased M1 macrophage and reduced the levels of miR-3061, which was negatively associated with Snail1 in RAW264.7 cells. Mechanistic studies demonstrated that miR-3061 regulated macrophage polarization by targeting the Snail1 mRNA 3′‐untranslated region. Moreover, miR-3061 overexpression suppressed Snail1 expression and inhibited M1 macrophage and inflammatory cytokines.

**Conclusion:**

This study elucidated that diabetes exacerbated sepsis-induced intestinal injury by promoting M1 macrophage polarization and further demonstrated that the miR-3061/Sani1 axis may be the potential target of macrophage polarization.

## Introduction

Sepsis is a life-threatening systemic inflammatory syndrome in response to infection, which is associated with a mortality rate of 40% or higher (1). Intestinal injury is the initiating factor of multiple organ dysfunction syndrome, contributing to the morbidity and mortality of sepsis (2). Diabetes increases the incidence of sepsis (3). For instance, epidemiological data of Spain have suggested that the annual incidence of sepsis increased from 76.5 to 113.3/100,000 population, which was higher for the population with diabetes (4). The glycolytic metabolic shift in diabetes is thought to cause deregulated innate and adaptive immune responses, causing sepsis uncontrolled spread of invading pathogens. Macrophages are thought to be the key players in diabetes progression and promote inflammation by proteases and pro-inflammatory cytokine release (5).

Macrophages exhibit distinctive phenotypic characters in response to diverse stimuli (6–8). Activated macrophages are usually polarized toward pro-inflammatory M1 and anti-inflammatory M2 phenotypes, and both categories are closely associated with inflammatory response (7). Macrophage polarization is considered the key factor in determining the progression of intestinal injury (9). M1 macrophages play important roles in sepsis-related tissue injury (10). Studies have shown that switching the polarized macrophages from M1 to M2 can effectively attenuate inflammation dysregulation and intestinal injury (11). Diabetic conditions favor the promotion of the M1 macrophage phenotype. The underlying mechanism may be the increased oxidative stress triggered by hyperglycemia (12). The reactive oxygen species (ROS) and reactive nitrogen species (RNS) caused by oxidative stress are important mediators for pro-inflammatory signaling pathway activation (13). In addition, M1 macrophage–associated inflammatory microenvironment is a well-recognized mechanism of diabetes progression (5, 14). Therefore, M1 macrophage may be the driven factor for developing sepsis in diabetes. However, the mechanisms of macrophage polarization in intestinal injury under diabetes and sepsis conditions are poorly understood.

MicroRNAs (miRNAs) are a class of 17- to 24-nt small non-coding RNAs and regulate the target messenger RNA (mRNA) by binding to 3′-untranslated regions (3′-UTR) (15). Accumulating studies have demonstrated that miRNAs are key regulators of macrophage polarization (16). For instance, miR-23a promotes macrophage polarization to the M2 phenotype by directly suppressing Toll-like receptor (TLR) and interferon (IFN) signaling (17). miR-27-3p regulates pro-inflammatory cytokine production in alveolar macrophage by targeting Peroxisome proliferator-activated receptor‐γ (PPAR‐γ) (18). miR-211 modulates M1 macrophage polarization *via* Signal transducer and activator of transcription 1 (STAT1); Signal transducer and activator of transcription 3 (STAT3); and Suppressor of cytokine signaling1 (SOCS1) (19). Hence, miRNAs play a key role in macrophage polarization through transcription factor regulation.

Snail1, the core transcription factor acting as an epithelial-to-mesenchymal transition (EMT) inducer, has been increasingly considered as a major regulator of macrophage activation. Snail1 has been involved in multiple cellular progresses including tumor invasion and migration, fibrosis, and inflammation (20–22). The question regarding whether or not Snail1 influences the inflammation microenvironment and macrophage polarization has been addressed in those related studies. For example, it is found that overexpression of Snail1 in lung cancer cells favors pro-inflammatory macrophage activation through ubiquitin-specific protease 4 (USP4) (23). On the other hand, Snail1 silence has been revealed to decrease the polarization of M1 macrophages and to alleviate the levels of inflammatory cytokines in the study on renal fibrosis (24). Under diabetic conditions, Snail1 expression seems to be particularly upregulated and promotes oxidative stress and inflammation (22). In addition, Snail1 silencing could effectively prevent the progression of diabetes-related complications (25). However, the connection between Snail1 and sepsis-induced intestinal injury under diabetic conditions is unknown.

Currently, clinical data have suggested that diabetes increases the incidence of sepsis, but little is known about the underlying molecular mechanisms. Here, we first identified the important role of macrophage polarization in diabetes-mediated aggravation of intestinal injury in sepsis. Further bioinformatic analysis and mechanistic studies revealed the key role of miR-3061 in macrophage polarization. Moreover, we demonstrated a vital post-transcriptional mechanism for Snail1 regulated by miR-3061 in the macrophage polarization–mediated sepsis-related intestinal injury in diabetic mice.

## Materials and methods

### Mice

Male wild-type (WT) (C57BL/KsJ) and diabetic (C57BL/KsJ db/db) mice aged 10–12 weeks were purchased from Guangdong Yaokang Biotechnology Co. (Foshan, China). All animal experiments received approval from the Institutional Animal Care and Use Committee of Sun Yat-Sen University (Guangzhou, Guangdong, China). Mice were housed in an specific-pathogen-free (SPF) barrier environment with 12-h light/dark cycle, relative humidity in the (55% + 5%), room temperature (23°C ± 2°C), and free access to water and food.

### CLP-induced sepsis model

Non-Diabetic (ND) and Diabetic (D) mice were randomly allocated to the following four groups to observe the hyperglycemia and sepsis-induced intestinal injury: ND sham group (NDMS group, n = 6), ND CLP group (NDMCLP group, n = 6), diabetic sham group (DMS group, n = 6), and diabetic CLP group (DMCLP group, n = 6). One percent sodium pentobarbital (50 mg/kg) was injected intraperitoneally to anesthetize the mice. After disinfection, the abdomen was incised 1 cm at midline to expose the cecum. Silk thread was subsequently used to ligate the proximal third of the cecum and then punctured once with a 22G needle, and fecal matter was extruded. Finally, the cecum was inserted back into abdomen, and the incision was sutured. After surgery, 4 ml/100 g of normal saline was injected subcutaneously for fluid resuscitation. In addition, bupivacaine and butorphanol tartrate were used for postoperative analgesia. For mice in the two sham groups, the cecum was only exposed and then returned to the abdomen. The serum and intestinal tissue were collected at 12 h after surgery.

### Macrophage depletion

The mechanism of macrophage depletion is described as follows. Clodronate liposome (Cls) is brought into the macrophages *via* an endocytosis mechanism. Then, lysosomes promote the release of clodronate dissolved in the liposomes. Clodronate can induce macrophage apoptosis, thus achieving the function of macrophage depletion.

Diabetic mice were randomly allocated to the following four groups to confirm the role of macrophage in sepsis-induced intestinal injury: phosphate buffered saline (PBS) + sham group (DMS group, n = 6), PBS + CLP group (DMCLP group, n = 6), Cls + sham group (DAMS group, n = 6), and Cls + CLP group (DAMCLP group, n = 6). The mice were intraperitoneally injected with 200 µl of Cls or control liposome (PBS) (YEASEN, Shanghai, China) 24 h later, and CLP was then performed. The detailed procedures were the same as abovementioned.

### Cell culture and treatment

RAW264.7 cells (obtained from the Cell Bank of the Chinese Academy of Science) were cultured in Dulbecco’s modified Eagle’s medium (KeyGen, China) supplemented with 10% fetal bovine serum (Gibco) and penicillin (100 U/ml) and streptomycin (100 μg/ml) (Gibco). Cells were cultured at 37°C in a humidified atmosphere with 5% CO_2_.

RAW264.7 cells were stimulated with 35 mM high glucose medium and lipopolysaccharide (LPS) (1 µg/ml; Sigma) for 24 h to establish the hyperglycemia and sepsis cell model.

### Histological assessment

Intestinal tissues were harvested at 5 cm above the terminal ileum. Tissues were fixed with 4% paraformaldehyde and embedded in paraffin. Consecutive tissue sections (5 µm) were stained with H&E. Chui’s scoring system was used to evaluate the intestinal mucosal damage (26): Grade 0, normal mucosa villi; Grade 1, development of the subepithelial Gruenhagen’s space at the tip of the villi; Grade 2, extension of the subepithelial space with moderate epithelial lifting; Grade 3, extensive epithelial lifting, possibly with a few denuded villi; Grade 4, denuded villi with dilated capillaries and increased cellularity of the lamina propria and exposed capillaries; and Grade 5, disintegration of the lamina propria, ulceration, and hemorrhage.

### Western blot analysis

Cells were harvested by Radio Immunoprecipitation Assay (RIPA) buffer (KeyGen). The protein concentration was measured using a bicinchoninic acid (BCA) protein assay kit (Thermo Fisher Scientific). Equivalent protein (30 μg) was separated by 4%–20% sodium dodecyl sulfate-polyacrylamide gel electrophoresis (SDS-PAGE) and transferred onto the polyvinylidene difluoride (PVDF) membrane. The membranes were blocked with 5% bovine serum albumin (BSA)/Tris-buffered saline–Tween-20 for 1 h at room temperature and then incubated with primary antibodies: anti-occludin (1:1,000, Abcam), anti-zonula occludens-1 (ZO-1) (1:1,000, ABclonal), anti-Snail1 (1:1,000, Cell Signaling Technology), and β-actin (1:10,000, ZO-1) (ABclonal) overnight at 4°C, followed by incubating with secondary antibodies (1:10,000, ABclonal) at room temperature for 1 h.

The images were obtained by Tanon 5500 imaging system (Tanon, Shanghai) and analyzed by ImageJ software.

### Enzyme linked immunosorbent assay (ELISA)

The serum samples were collected and stored at −80°C. The levels of LPS and intestinal fatty acid binding protein (iFABP) were measured with a mouse LPS and iFABP ELISA Kit (CUSABIO, Wuhan, China) according to the manufacturer’s instructions.

### Immunofluorescence

Frozen tissue sections and paraffin tissue sections were used for immunofluorescence staining. Paraffin sections were traditionally fixed, regularly dewaxed, repaired under high pressure, and closed. After sealing, samples were stained with the primary antibody: F4/80 (1:1,000, Cell Signaling Technology) at 4°C overnight, and then incubated with the fluorescent secondary antibody: anti-rabbit IgG-fluorescein isothiocyanate (IgG-FITC) (1:1,000, Cell Signaling Technology) according to the manufacturer’s instructions. All images were observed using a fluorescence microscope (EVOS FL, Life Technologies, Carlsbad, CA).

### Quantitative real-time PCR

Total RNA was extracted from intestinal tissue and RAW264.7 cells using a TRIzol reagent (Invitrogen, USA). Total RNA was converted to cDNA by TransScript II All-in-One First-Strand cDNA (GeneCopoeia, Guangzhou, China) for miRNA and ReverTra Ace qPCR RT Master Mix (Toyobo, Japan) for mRNA. Then, quantitative real-time PCR (qRT-PCR) was conducted using XX for miRNA and SYBR^®^ Green Real-time PCR Master Mix (TOYOBO, Japan) for mRNA. U6 and β-actin were used as the internal control for miRNA or mRNA. The primers were synthesized by Guangzhou IGE Biotechnology Corporation (Guangzhou, China), and the primer sequences for all genes are shown in **Tables S5, S6**. The 2^−ΔΔCt^ method was performed for quantitative analysis.

### RNA-seq and data analysis

The miRNA microarray assay was performed using the BGISEQ-500 platform by the BGI Company (Shenzhen, China). The raw data were acquired and normalized by TPM. Then, differentially expressed miRNAs were determined using DESeq2 by adjusting |fold change| ≥ 1.5 and p-value < 0.05. The heat map was achieved using R 3.5.3 software. The miRNA predicted target enrichment analysis using the TargetScan and miRDB database.

RNA-seq was constructed using Illumina HiSeq2000 by the BGI Company (Shenzhen, China). The mRNA expression in the isoform level was measured by transcript counts. Relative expression abundances were calculated in fragments per kilobase of transcript per million fragments mapped (FPKM) using Cufflinks. Differentially expressed mRNAs were also determined using DESeq2 by adjusting |fold change| ≥ 1.5 and p-value < 0.05. Immunocytic infiltration analysis was performed using xCell toll (https://xcell.ucsf.edu/).

### Dual-luciferase reporter assay

Dual-luciferase report assay was performed to test the direct binding ability between miR-3061 and Snail1. The 3′-UTR sequence of Snail1 containing the predicted binding sites and mutant sequence was cloned into a plasmid vector (GENE, China). miR-3061 overexpression plasmid or negative control (NC) and Snail1-wt or Snail1-mut plasmid were co-transfected into 293T cells by Lipofectamine 2000 (Invitrogen). The Renilla luciferase vector was co-transfected as the internal reference plasmid. Luciferase activity was detected by the Dual-Luciferase Reporter Assay System (Promega, USA).

### Cell transfection

Snail1 small interfering RNA (siRNA), miRNA-3061 mimics and inhibitors, as well as the corresponding NCs were synthesized by KeyGen Corporation (Nanjing, China). Lipofectamine 2000 (Invitrogen) was used for transfections following the manufacturer’s instructions.

### Statistical analysis

SPSS 13.0 (SPSS Inc., Chicago, IL, USA) and SigmaPlot 10.0 (Systat Software, Inc., Chicago, IL, USA) were used to perform statistical analysis. Normality of the data was tested using the Kolmogorov–Smirnov test. Multiple comparisons among different groups were analyzed using one-way analysis of variance, followed by Tukey’s *post hoc* test. Quantitative data are presented as means ± standard deviation. *P-*values less than 0.05 were considered statistically significant.

## Results

### Diabetes exacerbated sepsis-induced intestinal injury, and the damage severity was positively correlated with macrophage infiltration

**Figure 1:**
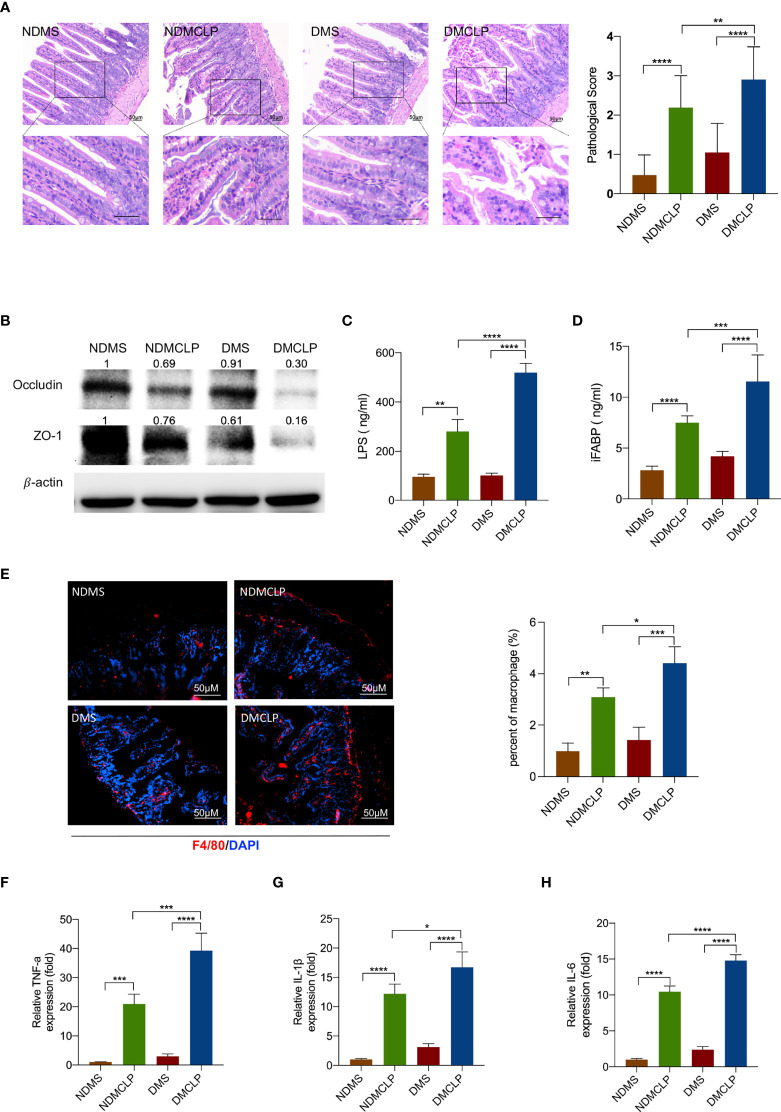
Diabetes exacerbated sepsis-induced intestinal injury and the damage severity positively correlated with macrophage infiltration. **(A)** H&E staining of intestinal tissue sections (200×) and pathological scores. **(B)** Expression of tight junction protein occludin and ZO-1 was measured by Western blotting. **(C, D)** Serum levels of intestinal mucosal barrier function biomarkers LPS and iFABP were measured by ELISA. **(E)** Immunofluorescence staining of F4/80 (red) in intestinal issues. **(F–H)** The levels of inflammatory cytokines in intestinal issues, including TNF-α, IL-1β, and IL-6, were measured by qPCR. Data were represented as the mean ± standard deviation. NDMS, sham group in WT mice; NDMCLP, the model group with CLP in WT mice; DMS, sham group in diabetic mice; DMCLP, CLP model group in diabetic mice. *p < 0.05, **p < 0.001, ***p < 0.001, and ****p < 0.0001.

Clinical data from literature search have indicated that diabetes increases the incidence of infection (**Table S1**), but little is known about the underlying molecular mechanisms. To observe the characteristics of sepsis-induced intestinal injury under diabetic condition, the cecal ligation and puncture (CLP) method was used to establish a sepsis model in ND WT (C57BL/KsJ) and diabetic (C57BL/KsJ db/db) mice. H&E staining showed that CLP caused markedly intestinal injury, and the injury score is significantly higher in diabetic CLP-mice than in ND WT mice (**Figure 1A**). The levels of tight junction proteins occludin and ZO-1 markedly decreased in septic mice, and these proteins were lower in the DMCLP group than in the NDMCLP group (**Figure 1B**). Under diabetes and sepsis conditions, the serum concentrations of LPS and iFABP were significantly augmented (**Figures 1C** **, D**).

To examine the macrophage infiltration of intestinal tissue, immunofluorescence analyses was utilized. It was observed that the macrophage marker F4/80+ was significantly increased in the DMCLP group compared with that in the NDMCLP group (**Figure 1E**). Then, qRT-PCR examination further revealed that the mRNA expression of both the M1 markers iNOS and CRR7 and the M2 markers Arg1 and Fizz1 were upregulated, with a more significant increase in M1 macrophages (**Figure S1**). The mRNA expression of cytokines including TNF-α, IL-1β, and IL-6 in intestinal tissue was also detected. Consistent with the trend with macrophage infiltration, the mRNA levels of these inflammatory cytokines were increased significantly (**Figures 1F** **–H**).

### Depletion of macrophages alleviated sepsis-induced intestinal injury in mice

**Figure 2:**
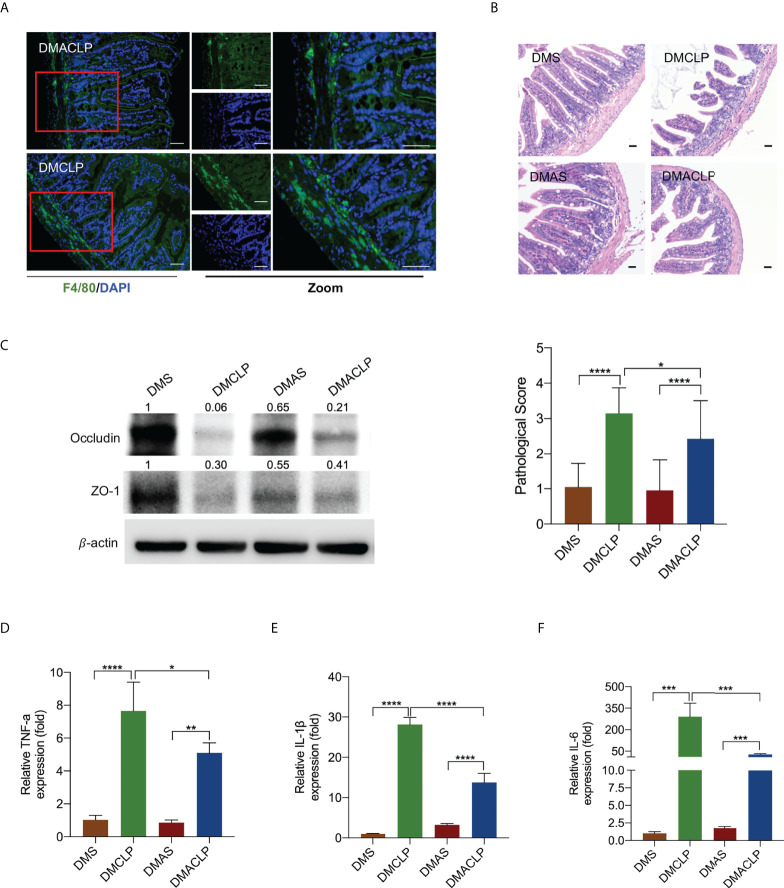
Decreased macrophages alleviated sepsis-induced intestinal injury in mice. **(A)** Immunofluorescence staining of F4/80 (green) in intestinal issues. **(B)** H&E staining of intestinal tissue sections (200×) and pathological scores. **(C)** Expression of tight junction protein occludin and ZO-1 was measured by Western blotting. **(D–F)** The levels of inflammatory cytokines in intestinal issues, including TNF-α, IL-1β, and IL-6, were measured by qPCR. Data were represented as the mean ± standard deviation. DMS, the diabetic mice were intraperitoneally injected with 200 µl of control liposome (PBS); DMCLP, the diabetic mice were intraperitoneally injected with 200 µl of control liposome (PBS) before CLP; DMAS, the diabetic mice were intraperitoneally injected with 200 µl of Clodronate liposome (Cls); DMACLP, the diabetic mice were intraperitoneally injected with 200 µl of Clodronate liposome (Cls) before CLP. *p < 0.05, **p < 0.001, ***p < 0.001, and ****p < 0.0001.

Next, we sought to confirm whether macrophages contribute to sepsis-induced intestinal injury under hyperglycemia condition. Cls was administered intraperitoneally to deplete macrophages in diabetic mice. Immunofluorescence analyses revealed that macrophage marker (F4/80+) dramatically decreased at 24 h after Cls treatment (**Figures 2A**, **S2**). qRT-PCR analysis confirmed that the mRNA expression of both the M1 markers iNOS and CRR7 and the M2 markers Arg1 and Fizze1 was significantly downregulated after Cls treatment (**Figure S3**). H&E staining showed that the injury scores were lower in macrophage-depleted diabetic mice (**Figure 2B**). Western blotting further showed that macrophage depletion significantly increased occludin and ZO-1 protein expression (**Figure 2C**). The levels of inflammatory cytokines TNF-α, IL-1β, and IL-6 were reduced substantially after Cls treatment (**Figures 2D** **–F**).

We further performed macrophage rescue experiments *in vitro*. We co-cultured hyperglycemia and LPS-induced M1 macrophages and intestinal mucosal epithelial cells. Flow cytometry analysis showed that hyperglycemia and LPS-stimulated M1 macrophages increased the apoptosis rate of intestinal mucosal epithelial cells compared with the control group (**Figure S4**), indicating that M1 macrophages aggravated intestinal injury under sepsis and diabetes condition.

### The decrease in miR-3061 was negatively associated with the level of M1 macrophage markers

**Figure 3:**
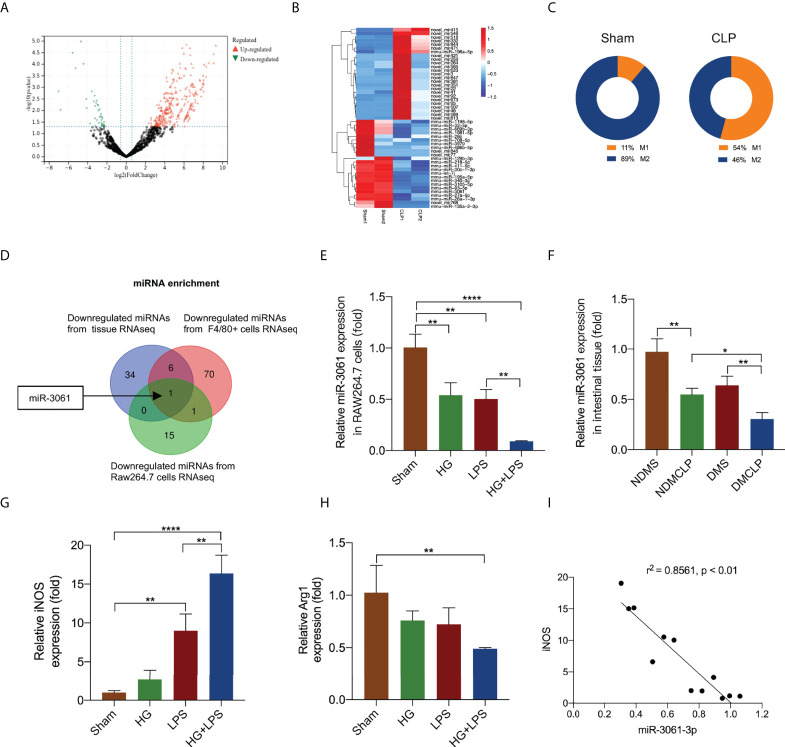
The decrease in miR-3061 was negatively associated with M1 macrophage markers. **(A)** The volcano plot of miRNAs. The red dots illustrate upregulated miRNAs, and the green dots display downregulated miRNAs. **(B)** Heat map of miRNA-seq. The fluorescence intensity of differentially expressed miRNAs (> 1.5 fold) is illustrated from high (red) to low (blue). **(C)** M1/M2 macrophages were detected by immunoinfiltration analysis. **(D)** Venn diagram of downregulated miRNAs in intestinal tissue RNA-seq and downregulated miRNAs in F4/80+ cells RNA-seq. **(E)** The expression of miR-3061-3p in intestinal tissue was detected by qRT-PCR. **(F)** The expression of miR-3061-3p was detected by qRT-PCR in RAW264.7 stimulated with HG + LPS. **(G, H)** The mRNA levels of macrophage markers iNOS and Arg1 were detected by qRT-PCR. **(I)** The correlation analysis about miR-3061-3p and M1 marker iNOS. *p < 0.05, **p < 0.001, and ****p < 0.0001.

To detect the miRNA expression, RNA-seq assay was performed in CLP-induced sepsis and normal intestinal tissue of diabetic mice. We identified 41 downregulated and 311 upregulated miRNAs, with negative or positive fold change greater than 1.5 (all *p* < 0.05, **Figure 3A** and **Table S2**). The partially differentially expressed miRNAs were depicted by a cluster heat map (**Figure 3B**). In addition, the immunoinfiltration analysis was utilized to verify the immune cell infiltration in septic intestinal tissue. M1/M2 macrophages were obviously increased (**Figure 3C** and **Table S3**). We further performed RNA-seq assay in HG + LPS–stimulated RAW264.7 cells and control cells. In addition, 17 downregulated and 36 upregulated miRNAs were identified, respectively, with negative or positive fold change greater than 1.5 (all *p* < 0.05, **Table S4**). The miRNAs were depicted by a cluster heat map (**Figure S5**). Furthermore, the downregulated miRNAs of intestinal tissue and RAW264.7 cells were used to overlap with the miRNA sequencing data from F4/80+ cells (**Table S5**) (27), and miR-3061 was identified as the potential target of macrophage polarization (**Figure 3D**). To confirm miR-3061 expression, we performed qRT-PCR analysis of HG + LPS–activated macrophages. The results revealed that the expression of miR-3061 was significantly decreased in both LPS and HG + LPS–activated macrophages (**Figure 3E**). The qRT-PCR analysis of the miR-3061 expression in intestinal tissue exhibited a consistent trend (**Figure 3F**). Moreover, we examined the polarization of RAW264.7 cells under high glucose and LPS stimulation. It was found that M1 marker iNOS expression significantly increased in the HG + LPS group compared with the LPS group, whereas the M2 macrophage marker Arg1 showed the opposite trend (**Figures 3G** **, H**). The correlation analysis revealed that miR-3061 is negatively correlated with iNOS (**Figure 3I**). These indicated that miR-3061 was negatively associated with M1 macrophage polarization.

### Enhancement of miR-3061 suppressed M1 macrophage polarization and the release of inflammatory factors

**Figure 4:**
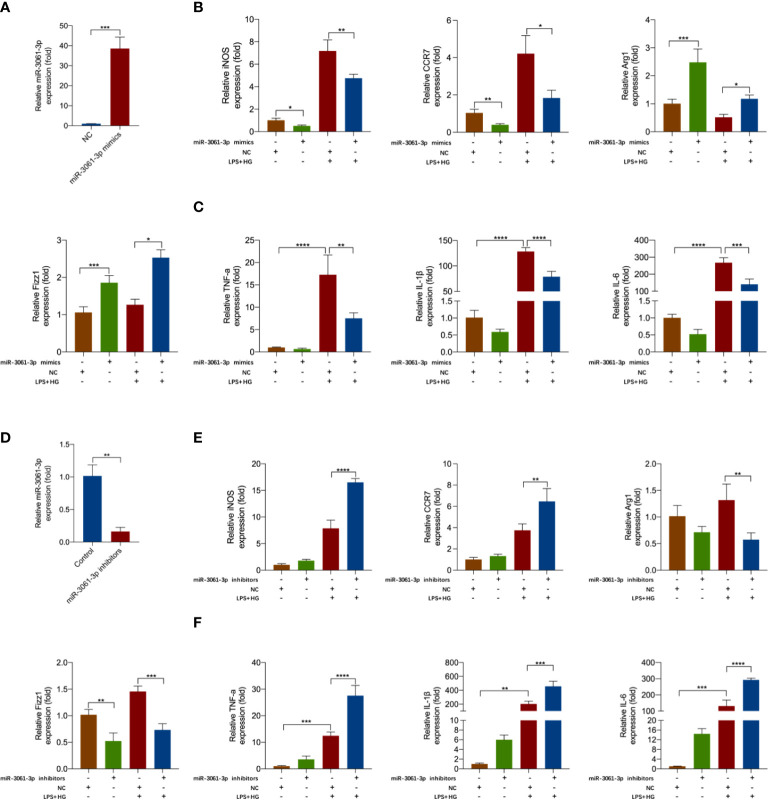
miR-3061-3p suppresses M1 macrophage polarization and inflammatory factors release. **(A)** The expression of miR-3061-3p in RAW264.7 cells transfected with mimics or NC was measured by qRT-PCR. **(B)** The mRNA levels of macrophage markers iNOS, CCR7, Arg1, and Fizz1 were detected by qRT-PCR in RAW264.7 cells transfected with mimics or NC. **(C)** The mRNA levels of inflammatory cytokines TNF-α, IL-1β, and IL-6 were measured by qPCR in RAW264.7 cells transfected with mimics or NC. **(D)** The expression of miR-3061-3p in RAW264.7 cells transfected with inhibitors or NC was measured by qRT-PCR. **(E)** The mRNA levels of macrophage markers iNOS, CCR7, Arg1, and Fizz1 were detected by qRT-PCR in RAW264.7 cells transfected with inhibitors or NC. **(F)** The mRNA levels of inflammatory cytokines TNF-α, IL-1β, and IL-6 were measured by qPCR in RAW264.7 cells transfected with inhibitors or NC. *p < 0.05, **p < 0.001, ***p < 0.001, and ****p < 0.0001.

To demonstrate the effect of miR-3061 in macrophage polarization, miR-3061 mimics and inhibitors were transfected into HG + LPS–activated RAW264.7 cells. We found that miR-3061 was significantly increased after miR-3061 mimic treatment compared with the NC group (**Figure 4A**). Moreover, the levels of M1 macrophage markers iNOS and CRR7 mRNA were significantly decreased in RAW264.7 cells transfected with miR-3061-3p mimics, whereas the M2 macrophage markers Arg1 and Fizz1 showed the opposite trend (**Figure 4B**). We also found that the inflammatory cytokines TNF-α, IL-1β, and IL-6 were reduced after miR-3061 was upregulated (**Figure 4C**).

To further study the effect of miR-3061 on the polarization of M1 macrophages, we transfected miR-3061 inhibitors into HG + LPS–activated RAW264.7 cells. As expected, miR-3061 was obviously decreased in the miR-3061 inhibitor group compared with the NC group (**Figure 4D**). It was found that the mRNA expression of iNOS and CRR7 was markedly upregulated after miR-3061 inhibition, whereas the opposite trend was found about M2 markers Arg1 and Fizz1 (**Figure 4E**). Furthermore, the data showed that TNF-α, IL-1β, and IL-6 were markedly increased in HG + LPS–activated RAW264.7 cells transfected with miR-3061 inhibitors (**Figure 4F**). These results indicated that upregulated miR‐3061 can inhibit the polarization of M1 macrophages and the release of inflammatory factors.

### Snail1 is a target gene of miR-3061

**Figure 5:**
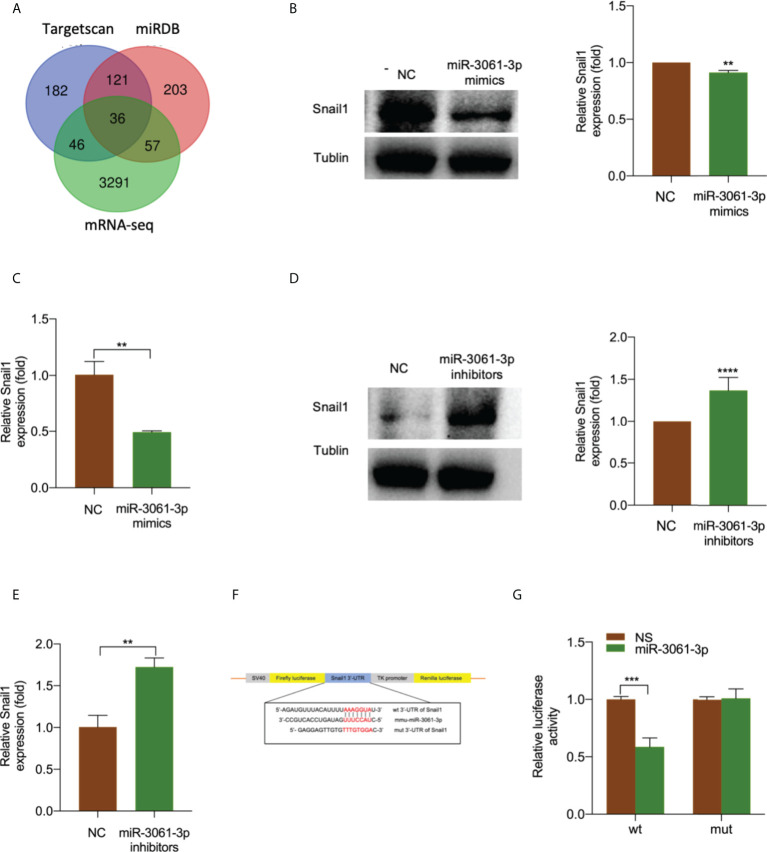
Snail1 is a target gene of miR-3061-3p. **(A)** Venn diagram of the predicted target genes of miR-3061 from TargetScan, miRDB, and mRNA-seq in intestinal tissue. **(B)** The protein expression of Snail1 in RAW264.7 cells transfected with miR-3061-3p mimics or NC was measured by Western blotting. **(C)** The mRNA expression of Snail1 in RAW264.7 cells transfected with miR-3061-3p mimics or NC was measured by qRT-PCR. **(D)** The protein expression of Snail1 in RAW264.7 cells transfected with miR-3061-3p inhibitors or NC was measured by Western blotting. **(E)** The mRNA expression of Snail1 in RAW264.7 cells transfected with miR-3061-3p inhibitors or NC was measured by qRT-PCR. **(F)** Binding sites of the Snail1 3′-UTR to miR-3-61-3p. **(G)** Luciferase activity of wild type (WT) and mutant type (MUT) of Snail1 detected by a luciferase reporter assay. **p < 0.001, ***p < 0.001, and ****p < 0.0001.

miR-3061 target enrichment analysis using TargetScan, miRDB database, and mRNA sequencing data identified 36 genes (**Figures 5A**, **S6**). Furthermore, we examined the predicted target mRNAs expression levels in the small intestinal tissue and monocyte cells using The Human Protein Atlas at https://www.proteinatlas.org. Of these candidate mRNAs, Snail1 expression was the highest (**Figures S7, S8**). Western blot and qRT-PCR analyses were performed to confirm the effect of miR-3061 on Snail1 expression. In addition, both mRNA and protein expressions of Snail1 were significantly decreased in the miR-3061 mimic group compared with the NC group, whereas the opposite trend was found in the miR-3061 inhibitor group (**Figures 5B** **–E**). According to the prediction result of TargetScan and miRDB database, there is a binding site between miR-3061 and Snail1 mRNA 3′-UTR (**Figure S9**). The dual-luciferase reporter assay was used to verify the binding sites. We constructed plasmids containing WT and mutant-type Snail1 mRNA 3′-UTR (**Figure 5F**). It was found that the luciferase activity of the WT Snail1 plasmid was markedly suppressed by miR-3061 compared with the NC group, whereas it was unchanged in the mutant-type Snail1 plasmid (**Figure 5G**). These data demonstrated that miR-3061 can directly target Snail1 by binding to its 3′-UTR.

### Hyperglycemia and LPS upregulated Snail1 expression to promote M1 macrophage polarization

**Figure 6:**
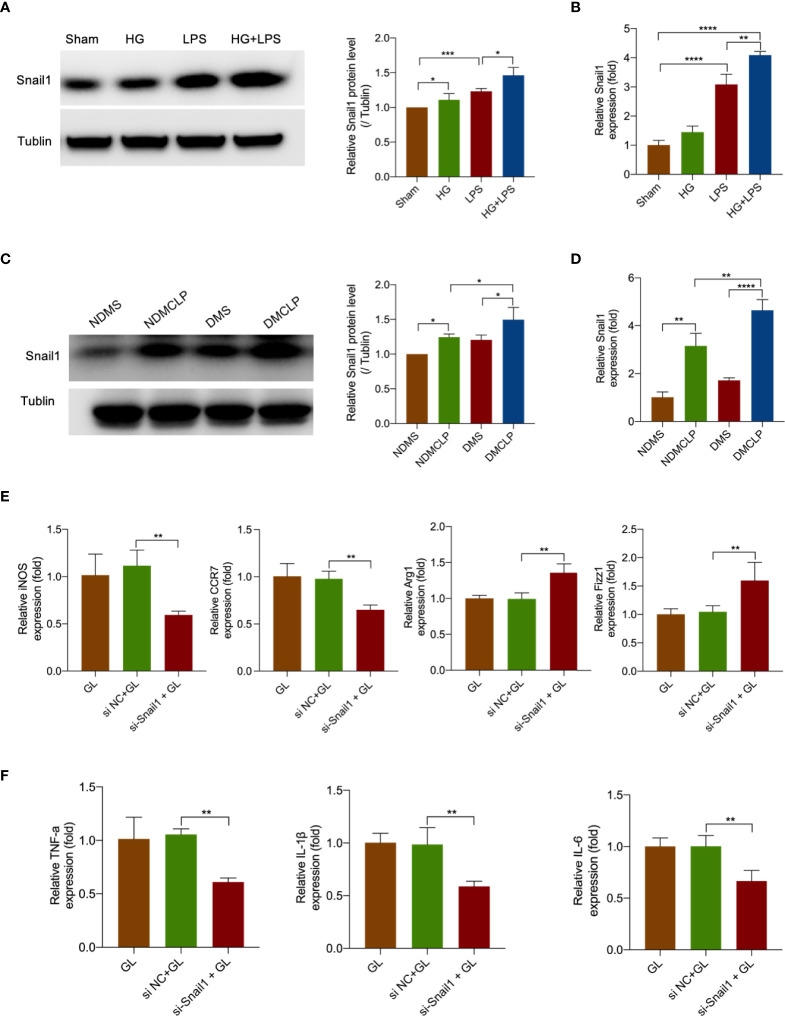
Hyperglycemia and LPS upregulated Snail1 expression to promote M1 macrophage polarization. **(A)** The protein expression of Snail1 in RAW264.7 stimulated with HG + LPS was measured by Western blotting. **(B)** The mRNA expression of Snail1 in RAW264.7 stimulated with HG + LPS was detected by qRT-PCR. **(C)** The protein expression of Snail1 in intestinal tissue was measured by Western blotting. **(D)** The mRNA expression of Snail1 in intestinal tissue was detected by qRT-PCR. **(E)** The mRNA levels of macrophage markers iNOS, CCR7, Arg1, and Fizz1 were detected by qRT-PCR in RAW264.7 cells transfected with Snail1 siRNA or NC. **(F)** The mRNA levels of inflammatory cytokines TNF-α, IL-1β, and IL-6 were measured by qPCR in RAW264.7 cells transfected with Snail1 siRNA or NC. *p < 0.05, **p < 0.001, ***p < 0.001, and ****p < 0.0001.

To examine the expression of transcription factor Snail1 in sepsis-induced intestinal tissue and HG + LPS–activated RAW264.7 cells, Western blot and qRT-PCR analyses were performed. It was found that Snail1 protein and mRNA levels were significantly increased in both tissue and cells (**Figures 6A** **–D**).

To further research the effect of Snail1 in macrophage polarization, we transfected Snail1 siRNA in HG + LPS–activated RAW264.7 cells. As expected, Snail1 mRNA was markedly reduced in the si-Snail1 group compared with the NC group (**Figure S10**). Moreover, M1 macrophage markers including iNOS and CRR7 were obviously inhibited in the si-Snail1 group compared with the NC group (**Figure 6E**). Furthermore, the data showed that the inflammatory cytokines TNF-α, IL-1β, and IL-6 were also reduced after Snail1 was being downregulated (**Figure 6F**). These data indicated that decreased Snail1 expression could effectively suppress M1 macrophage polarization.

### Snail1 knockdown reversed the effects of miR-3061 inhibition on M1 macrophage polarization

**Figure 7:**
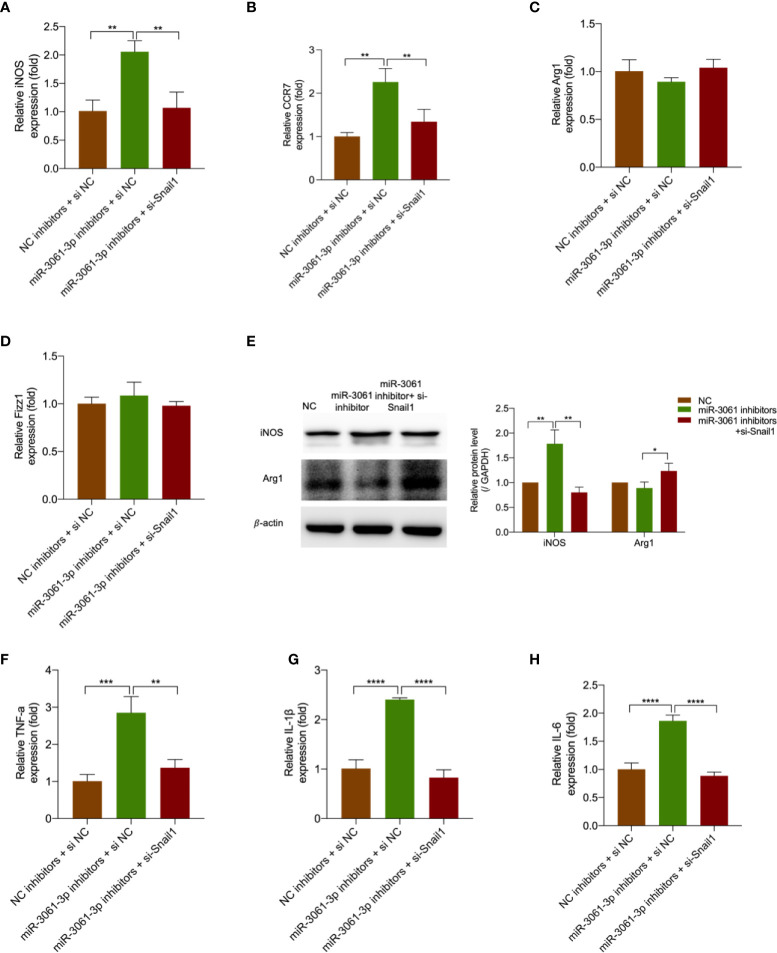
Decreased Snail1 reversed the promoting effects of miR-3061 inhibitors. **(A–D)** The mRNA levels of macrophage markers iNOS, CCR7, Arg1, and Fizz1 were detected by qRT-PCR in RAW264.7 cells transfected with miR-3061-3p inhibitors and Snail1 siRNA. **(E)** The effect of downregulated miR-3061-3p and Snail1 on iNOS and Arg1 protein expression was detected by Western blotting. **(F–H)** The mRNA levels of inflammatory cytokines TNF-α, IL-1β, and IL-6 were measured by qPCR in RAW264.7 cells transfected with miR-3061-3p inhibitors and Snail1 siRNA. *p < 0.05, **p < 0.001, ***p < 0.001, and ****p < 0.0001.

To future verify the effect of miR-3061 in Snail1-mediated M1 macrophage polarization, miR-3061 inhibitors and Snail1 siRNA were transfected into HG + LPS–activated RAW264.7 cells. It was found that the upregulation of iNOS and CRR7 caused by miR-3061 inhibitors was markedly reversed after Snail1 siRNA transfection (**Figures 7A** **, B**), whereas the M2 markers Arg1 and Fizz1 were not significantly changed (**Figures 7C** **, D**). The Western blot assay data showed that iNOS and Arg1 protein expression trends were consistent with the results by RT-qPCR (**Figure 7E**). Moreover, the results showed that the inflammatory cytokines TNF-α, IL-1β, and IL-6 were also increased with the use of miR-3061 inhibitors and significantly reduced by Snail1 siRNA transfection (**Figures 7F** **–H**). The results indicated that silencing Snail1 can reverse the effect of miR-3061 inhibitors in promoting M1 macrophage polarization.

## Discussion

Clinical data have suggested that diabetes increases the incidence of sepsis, but little is known about the underlying molecular mechanisms. The present study first demonstrated the prominent role of M1 macrophages in sepsis-related intestinal tissue in diabetic mice. Further bioinformatics analysis and mechanistic studies found a previously unidentified role of miR-3061 in macrophage polarization under diabetes and sepsis conditions. The decrease in miR-3061 induced M1 macrophage polarization and promoted the release of inflammatory by targeting Snail1.

miRNAs have been considered as critical regulators of macrophage polarization (16). Multiple studies have shown that miRNA expression profiles may be altered in macrophages under M1- and M2-polarized conditions (28, 29). Moreover, the exact role of miRNAs in macrophage polarization is continuously being elucidated. For instance, miR-23a promotes macrophage polarization to the M2 phenotype by directly suppressing TLR and IFN signaling (17). miR-27-3p enhances M1 polarization and promotes pro-inflammatory cytokine production in alveolar macrophage by targeting Peroxisome proliferator-activated receptor‐γ (PPAR‐γ) (18). miR-155 may modulate M1 macrophage polarization *via* the JNK, Akt, and STAT6 pathway (30, 31). Here, in our study, miR-3061 was shown to be the functional molecule in macrophage polarization *via* RNA-seq assay and literature search. miR-3061-3p is highly evolutionarily conserved in humans and mice, which is located on human chromosomes 22 and mouse chromosomes 16, respectively. Our study discovered the new role of miR-3061 in M1 polarization of macrophages, which might be a novel mechanism in sepsis-related intestinal injury in diabetic mice. In our array analysis, it was confirmed that miR-3061 was decreased in sepsis-related intestinal tissues. We further discovered that the expression level of miR-3061 was significantly decreased in LPS-stimulated M1 macrophages. However, the precise mechanisms of miR-3061 regulated macrophage polarization need to be further addressed.

It is considered that transcription factors are fully involved in macrophage activation (32, 33). Recent studies have shown that the key transcription factor Snail1 is essential for macrophage polarization and metastasis in breast cancer through altered granulocyte-macrophage colony-stimulating factor (GM-CSF) secretion (34). Overexpression of Snail1 in lung cancer cells shows increased production of inflammatory cytokines TNF-α, IL-1β, and IL-6 by regulating macrophage activation (23). Snail1 silence has also been revealed to decrease the polarization of macrophages and alleviate the levels of inflammatory cytokines in the study on renal fibrosis (24). These studies verify that Snail1 is closely associated with M1 macrophage polarization. In the current study, it was found that Snail1 expression was significantly increased in both LPS-induced M1 macrophages and sepsis-related intestinal tissues in diabetic mice. Application of Snail1 siRNA in RAW264.7 cells significantly inhibited the M1 macrophage and the production of TNF-α, IL-1β, and IL-6. However, little is known about the epigenetic mechanisms of the miRNA/Snail1 axis in regulating macrophage polarization.

Macrophages are considered to be the crucial innate immune cells and play the prominent role in the pathogenesis of sepsis-induced intestinal injury (35, 36). The resident macrophages located below the intestinal mucosa exhibit a distinctive phenotypic character in different gut homeostatic conditions (9). The pro-inflammatory M1 macrophages increased in the early stage of sepsis, causing tissue damage through the production of inflammatory cytokines, chemokines, NO, and ROS (5, 37). However, little is known about the mechanism of macrophage polarization in diabetes and sepsis-induced intestinal injury. Here, our results showed that diabetes exacerbated sepsis-induced intestinal injury and that the damage severity positively correlated with M1 macrophage polarization. We further demonstrated that depletion of macrophages alleviated sepsis-induced intestinal injury in diabetic mice, suggesting that the damage mechanism might be attributable to pro-inflammatory M1 macrophage polarization in diabetic mice.

Two limitations were exhibited in our study. On one hand, only the CLP-induced sepsis animal model is performed in our study. LPS and CLP are considered the conventional methods for experimental animal models of sepsis. It has been demonstrated that the two models have similar mortality. However, the expression levels of LPS-induced cytokines (TNF, IL-6, IL-1, chemokines, and chemokines and macrophage inflammatory protein-2, MIP-2) peak between 1.5 and 4 h and decline at 8 h. In contrast, the expression levels of cytokines continue to increase at 8 h in the CLP model (38). Hence, the CLP-induced sepsis model does accurately mimic the cytokine profile of sepsis. On the other hand, there is a lack of epidemiological data about diabetes-mediated exacerbation of sepsis-induced intestinal injury in the present study. Moreover, evidence about corrections of miR-3061 with clinical intestinal mucosal barrier function indicators such as LDH and iFABP is needed to make the current conclusion more clinically relevant.

## Conclusions

In conclusion, our study demonstrated that diabetes exacerbated sepsis-induced intestinal injury by promoting M1 macrophage polarization. Mechanistically, our data suggested the involvement of the miR-3061-3p/Snail1 axis in promoting M1 macrophage polarization. These results may lead to a better understanding of the potential epigenetic mechanisms of sepsis-induced intestinal injury in diabetic conditions.

## Data availability statement

The datasets presented in this study can be found in online repositories. The name of the repository and accession number can be found below: NCBI Gene Expression Omnibus; GSE202261.

## Ethics statement

The animal study was reviewed and approved by the Institutional Animal Care and Use Committee of Sun Yat-Sen University.

## Author contributions

XC, NL, and ZX conceived of the study. Data were generated and analyzed by FT, YC, and TW. LZ, SZ, JC, CP, and WX provided clinical expertise to the study design or data interpretation. All authors contributed to the article and approved the submitted version.

## Funding

This work was supported by the National Natural Science Foundation of China (Nos. 82070072 and 81873644) for XC.

## Acknowledgments

We would like to thank the staff at the Department of Anesthesiology, The Third Affiliated Hospital of Sun Yat-Sen University, Guangzhou City, People’s Republic of China. The authors acknowledge VanScholar Editors Co., Ltd., Vancouver, Canada, for the English editing service.

## Conflict of interest

The authors declare that the research was conducted in the absence of any commercial or financial relationships that could be construed as a potential conflict of interest.

## Publisher’s note

All claims expressed in this article are solely those of the authors and do not necessarily represent those of their affiliated organizations, or those of the publisher, the editors and the reviewers. Any product that may be evaluated in this article, or claim that may be made by its manufacturer, is not guaranteed or endorsed by the publisher.

## Abbreviations

CLP, cecal ligation and puncture; WT, wild type; Cls, clodronate liposome; ROS, reactive oxygen species; RNS, reactive nitrogen species; EMT, epithelial-to-mesenchymal transition; USP4, ubiquitin-specific protease 4; BCA, bicinchoninic acid; PVDF, polyvinylidene difluoride; iFABP, intestinal fatty acid binding protein; qRT-PCR, quantitative real-time PCR; siRNA, small interfering RNA; LPS, lipopolysaccharide.
