# Supplementary material for: Diabetes exacerbated sepsis-induced intestinal injury by promoting M1 macrophage polarization via miR-3061/Snail1 signaling
Source: Front Immunol. 2022 Sep 9;13:922614. doi: 10.3389/fimmu.2022.922614 (PMC9503829; doi:10.3389/fimmu.2022.922614)
Supplement: Supplementary file 1 [file DataSheet_1.docx]

**Supplemental Tables**

**Table S1. Literatures about diabetes increases the incidence of infection**

| Ref | Patient Numbers | year | Infection (%) | |
| --- | --- | --- | --- | --- |
|  |  |  | diabetes | Non-diabetes |
| [1] | 113 | 2000 | 31% | 23% |
| [2] | 7418 | 2006 | 16.6% | 11.1% |
| [3] | 206 | 2009 | 30.7% | 11% |
| [4] | 1104 | 2016 | 17.6% | 9.8% |
| [5] | 41492 | 2017 | 10.7% | 7.1% |
| [6] | 1527 | 2021 | 39.7% | 24.2% |

**Table S2. Upregulated and downregulated miRNAs in intestinal tissue (|Fold Change|>1.5, p>0.05)**

| miRNAs | log2FoldChange | P value | miRNAs | log2FoldChange | P value |
| --- | --- | --- | --- | --- | --- |
| mmu-let-7f-1-3p | 3.559598339 | 0.017221474 | novel_mir332 | 7.970328767 | 0.000302452 |
| mmu-let-7f-2-3p | -2.512832207 | 0.044435367 | novel_mir335 | 2.99237639 | 0.048203463 |
| mmu-let-7j | -4.999848132 | 2.03E-06 | novel_mir347 | 4.169004416 | 0.002963863 |
| mmu-miR-10b-5p | -2.63099298 | 0.014463277 | novel_mir351 | 7.79236182 | 0.001207032 |
| mmu-miR-1187 | 2.600767995 | 0.043478822 | novel_mir356 | 3.143553162 | 0.046078841 |
| mmu-miR-1192 | 4.687681925 | 0.004812055 | novel_mir359 | 5.57318903 | 0.011238131 |
| mmu-miR-1195 | 4.233577193 | 0.000547493 | novel_mir362 | 4.866230204 | 0.016991445 |
| mmu-miR-1198-5p | -3.240224274 | 0.006582524 | novel_mir381 | 8.047607246 | 0.00071915 |
| mmu-miR-122-5p | 3.341581823 | 0.008002351 | novel_mir383 | 6.555661589 | 0.009024839 |
| mmu-miR-129b-3p | -4.116283965 | 0.008206041 | novel_mir386 | 4.320190343 | 0.012844979 |
| mmu-miR-133b-3p | 2.578659769 | 0.020243433 | novel_mir387 | 7.150853532 | 0.004354731 |
| mmu-miR-135a-2-3p | -5.569338953 | 3.34E-05 | novel_mir39 | 7.628509129 | 0.000460844 |
| mmu-miR-146a-5p | -2.57636328 | 0.036776179 | novel_mir391 | 4.542304788 | 0.000951795 |
| mmu-miR-154-5p | -2.655550028 | 0.018302224 | novel_mir393 | 6.277573143 | 0.019449932 |
| mmu-miR-181a-1-3p | -2.343441855 | 0.045513587 | novel_mir395 | 6.60010044 | 0.020137055 |
| mmu-miR-184-3p | -2.297626151 | 0.047702843 | novel_mir396 | 4.198126728 | 0.012152559 |
| mmu-miR-1901 | 6.406638864 | 0.010853821 | novel_mir399 | 4.407705973 | 0.006676992 |
| mmu-miR-194-5p | -2.489774868 | 0.022327222 | novel_mir4 | 3.935196479 | 0.001886404 |
| mmu-miR-1946a | 2.74161913 | 0.048374165 | novel_mir400 | 3.828784112 | 0.023740762 |
| mmu-miR-1946b | 4.182102182 | 0.000368105 | novel_mir409 | 6.27200547 | 0.014960663 |
| mmu-miR-1949 | 2.891841363 | 0.027082959 | novel_mir41 | 8.499707535 | 1.86E-06 |
| mmu-miR-195a-5p | -3.059321114 | 0.00387203 | novel_mir411 | 5.52801646 | 0.000363861 |
| mmu-miR-1961 | 6.2583713 | 1.40E-05 | novel_mir414 | 4.662571766 | 0.001753458 |
| mmu-miR-196a-1-3p | 3.490063416 | 0.025366215 | novel_mir415 | 7.671176221 | 0.00049071 |
| mmu-miR-196a-2-3p | 4.876720123 | 0.000181712 | novel_mir416 | 6.801780469 | 0.00852895 |
| mmu-miR-196a-5p | 8.449437935 | 1.30E-09 | novel_mir42 | 5.529016053 | 0.00039694 |
| mmu-miR-1981-5p | -2.902052604 | 0.010266631 | novel_mir421 | 7.777475926 | 0.002392942 |
| mmu-miR-200b-5p | -2.298929972 | 0.032570413 | novel_mir422 | 4.885004452 | 0.04131197 |
| mmu-miR-2137 | 4.809898319 | 0.000626886 | novel_mir423 | 6.284683534 | 0.002397188 |
| mmu-miR-218-5p | -4.436700842 | 9.95E-05 | novel_mir427 | 7.275296923 | 0.00188847 |
| mmu-miR-21a-5p | -2.757150674 | 0.009101643 | novel_mir430 | 6.951609768 | 0.00641033 |
| mmu-miR-224-3p | -2.479624397 | 0.048389475 | novel_mir431 | 5.221284623 | 0.001655181 |
| mmu-miR-26a-1-3p | -3.277764865 | 0.002363248 | novel_mir433 | 6.506360447 | 0.023535182 |
| mmu-miR-27a-5p | -5.25772384 | 0.00015456 | novel_mir434 | 6.724353997 | 0.000765327 |
| mmu-miR-28c | -3.755664433 | 0.038525359 | novel_mir435 | 3.530114477 | 0.049669151 |
| mmu-miR-29b-1-5p | -2.583056919 | 0.027476507 | novel_mir454 | 6.777132236 | 0.005931248 |
| mmu-miR-3061 | -3.054913763 | 0.026938289 | novel_mir455 | 3.631299303 | 0.010109709 |
| mmu-miR-3074-1-3p | 3.128519617 | 0.01973052 | novel_mir458 | 4.579051836 | 0.002708048 |
| mmu-miR-30c-1-3p | -2.944421053 | 0.006221796 | novel_mir46 | 8.041344541 | 0.000471053 |
| mmu-miR-3105-5p | -3.124512856 | 0.005020442 | novel_mir468 | 3.28905513 | 0.035531909 |
| mmu-miR-32-5p | -3.360482824 | 0.004171966 | novel_mir47 | 4.516437005 | 0.036139592 |
| mmu-miR-340-3p | -2.917486328 | 0.005896166 | novel_mir470 | 3.964470621 | 0.00262951 |
| mmu-miR-3470a | 3.57436018 | 0.003726404 | novel_mir471 | 8.443315165 | 3.61E-10 |
| mmu-miR-3470b | 3.414256496 | 0.005440561 | novel_mir474 | 6.013712027 | 0.02787931 |
| mmu-miR-3471 | 4.179200485 | 0.001624761 | novel_mir480 | 5.035661914 | 0.048980139 |
| mmu-miR-3472 | 4.249311798 | 0.008872942 | novel_mir481 | 4.118104677 | 0.008991642 |
| mmu-miR-3473a | 5.967207353 | 3.45E-05 | novel_mir482 | 5.8223316 | 1.95E-05 |
| mmu-miR-3473b | 4.074973274 | 0.004054184 | novel_mir484 | 4.079213106 | 0.02797431 |
| mmu-miR-3473c | 5.554199223 | 9.12E-05 | novel_mir486 | 4.733381249 | 0.002861765 |
| mmu-miR-3473f | 3.473211594 | 0.007320333 | novel_mir49 | 6.299984003 | 0.000993268 |
| mmu-miR-34a-5p | -2.754251954 | 0.019816932 | novel_mir491 | 4.674367192 | 0.009462298 |
| mmu-miR-379-3p | -2.419326774 | 0.039662901 | novel_mir493 | 7.183204944 | 0.00172321 |
| mmu-miR-384-3p | -2.510336072 | 0.019855809 | novel_mir494 | 6.550102769 | 0.001316895 |
| mmu-miR-3970 | -3.221960291 | 0.047252222 | novel_mir496 | 3.032867758 | 0.036137023 |
| mmu-miR-411-5p | -3.568539922 | 0.001539063 | novel_mir500 | 4.261394375 | 0.001751176 |
| mmu-miR-466k | 2.585554132 | 0.034446686 | novel_mir501 | 6.356717433 | 0.013573794 |
| mmu-miR-467c-5p | -2.269691175 | 0.045836035 | novel_mir503 | 3.182239858 | 0.005637442 |
| mmu-miR-467e-5p | -2.388386522 | 0.049710941 | novel_mir507 | 8.276012296 | 0.000342691 |
| mmu-miR-486b-5p | -3.248923444 | 0.022529621 | novel_mir510 | 4.559762878 | 0.007220723 |
| mmu-miR-5100 | 3.125081478 | 0.034607171 | novel_mir516 | 11.11456907 | 6.87E-11 |
| mmu-miR-5121 | 4.026949528 | 0.001745773 | novel_mir522 | 7.061142184 | 0.002113829 |
| mmu-miR-5126 | 4.943307701 | 0.000431652 | novel_mir523 | 3.603175628 | 0.036237558 |
| mmu-miR-6236 | 3.685028015 | 0.009438248 | novel_mir526 | 3.019963432 | 0.024850626 |
| mmu-miR-6239 | 5.294515015 | 0.005594243 | novel_mir529 | 3.413286036 | 0.016815316 |
| mmu-miR-6240 | 3.652771347 | 0.007092577 | novel_mir534 | 4.560396044 | 0.007579965 |
| mmu-miR-6538 | 6.602193545 | 1.95E-05 | novel_mir535 | 3.124981181 | 0.043342882 |
| mmu-miR-669b-3p | -4.153780857 | 0.003365018 | novel_mir54 | 3.745510798 | 0.010562059 |
| mmu-miR-669p-3p | 3.446806299 | 0.023245157 | novel_mir544 | 3.973579356 | 0.042036894 |
| mmu-miR-682 | 3.241797719 | 0.024452552 | novel_mir546 | 8.093003934 | 0.000103703 |
| mmu-miR-690 | 2.658986028 | 0.049479728 | novel_mir549 | 3.482979979 | 0.016031744 |
| mmu-miR-692 | 3.03896543 | 0.026468605 | novel_mir55 | 5.076371263 | 0.00168637 |
| mmu-miR-696 | 4.847561758 | 0.000438274 | novel_mir552 | 5.341198773 | 0.005266969 |
| mmu-miR-703 | 3.422439241 | 0.034742958 | novel_mir555 | 4.300087679 | 0.008079723 |
| mmu-miR-7033-3p | 3.770750018 | 0.030130549 | novel_mir558 | 6.954478019 | 0.000138342 |
| mmu-miR-7033-5p | 3.926790773 | 0.022578455 | novel_mir559 | 6.182470632 | 0.003153469 |
| mmu-miR-706 | 4.732525628 | 0.000231255 | novel_mir56 | 4.07034188 | 0.00977648 |
| mmu-miR-708-5p | -2.84418262 | 0.036208807 | novel_mir560 | 4.109808054 | 0.033988522 |
| mmu-miR-709 | 4.784332314 | 0.000163349 | novel_mir564 | 5.45670882 | 0.014169903 |
| mmu-miR-712-3p | 4.584132661 | 0.000944641 | novel_mir565 | 8.553397379 | 8.57E-05 |
| mmu-miR-712-5p | 5.38543406 | 0.004790357 | novel_mir566 | 6.650623554 | 3.90E-05 |
| mmu-miR-714 | 3.965170926 | 0.012404422 | novel_mir572 | 4.486253097 | 0.004699932 |
| mmu-miR-8111 | 2.58564797 | 0.02423168 | novel_mir580 | 3.783740987 | 0.044254696 |
| mmu-miR-8117 | 6.445974784 | 0.016408021 | novel_mir589 | 7.685031031 | 0.001182993 |
| mmu-miR-877-5p | -2.553589697 | 0.045547204 | novel_mir595 | 6.110192523 | 0.021258688 |
| mmu-miR-93-5p | -4.701676717 | 1.08E-05 | novel_mir600 | 3.558912009 | 0.032734501 |
| mmu-miR-99a-3p | 2.323186612 | 0.034244516 | novel_mir605 | 4.037441625 | 0.01247527 |
| novel_mir102 | 6.834930587 | 0.00067351 | novel_mir606 | 3.080895617 | 0.028490563 |
| novel_mir103 | 6.000731637 | 0.000306512 | novel_mir61 | 4.315470813 | 0.003803553 |
| novel_mir104 | 5.191994278 | 0.005511943 | novel_mir617 | 5.136690037 | 0.001433876 |
| novel_mir108 | 4.61430527 | 0.019259087 | novel_mir618 | 5.434664243 | 0.014824749 |
| novel_mir11 | 6.645825267 | 0.007469092 | novel_mir623 | 9.298013585 | 1.52E-05 |
| novel_mir122 | 4.028687527 | 0.044514668 | novel_mir628 | 6.654381217 | 0.00164491 |
| novel_mir125 | 5.665095396 | 0.009279369 | novel_mir63 | 4.514569654 | 0.005579969 |
| novel_mir127 | 4.794905908 | 0.003441766 | novel_mir630 | 3.682358219 | 0.023045184 |
| novel_mir131 | 7.10499011 | 0.000935474 | novel_mir636 | 3.822779392 | 0.042103492 |
| novel_mir136 | 4.245057549 | 0.033913727 | novel_mir64 | 3.984633434 | 0.009960564 |
| novel_mir138 | 6.735929921 | 0.004910563 | novel_mir642 | 5.004513735 | 0.036185876 |
| novel_mir139 | 5.255393606 | 0.030234127 | novel_mir643 | 3.560472108 | 0.010082334 |
| novel_mir145 | 4.090612274 | 0.014382646 | novel_mir644 | 3.626412402 | 0.007301433 |
| novel_mir146 | 4.479859609 | 0.002725767 | novel_mir65 | 6.277573143 | 0.019449932 |
| novel_mir15 | 4.954761451 | 0.000516001 | novel_mir654 | 5.484353338 | 9.73E-06 |
| novel_mir153 | 6.123089949 | 0.000142314 | novel_mir655 | 3.863139352 | 0.016298599 |
| novel_mir155 | 6.445974784 | 0.016408021 | novel_mir656 | 3.635831462 | 0.028015294 |
| novel_mir159 | 6.412011504 | 0.004010879 | novel_mir66 | 3.143376869 | 0.031399241 |
| novel_mir16 | 3.653151222 | 0.019748301 | novel_mir660 | 3.464152561 | 0.019866848 |
| novel_mir160 | 4.107132132 | 0.012647292 | novel_mir666 | 4.029151947 | 0.027728445 |
| novel_mir163 | 4.662136279 | 0.01091681 | novel_mir668 | 3.61115944 | 0.009498471 |
| novel_mir165 | 6.920689152 | 0.000941928 | novel_mir669 | 5.36330328 | 0.017539367 |
| novel_mir166 | 4.447272674 | 0.01908312 | novel_mir673 | 9.947218558 | 9.88E-10 |
| novel_mir17 | 3.274447537 | 0.041617275 | novel_mir675 | 4.640273602 | 0.003817355 |
| novel_mir170 | 3.120982498 | 0.047186967 | novel_mir679 | 7.062607238 | 0.008996461 |
| novel_mir173 | 5.268050181 | 0.00050761 | novel_mir683 | 6.995050597 | 0.010153541 |
| novel_mir175 | 4.416676939 | 0.00175366 | novel_mir684 | 3.720400462 | 0.036573036 |
| novel_mir177 | 6.897880708 | 0.004899927 | novel_mir685 | 4.915575871 | 0.00278507 |
| novel_mir179 | 4.146952493 | 0.013422335 | novel_mir687 | 3.351737859 | 0.038670607 |
| novel_mir185 | 6.700041718 | 0.005371185 | novel_mir694 | 6.671350308 | 0.006420605 |
| novel_mir189 | 3.044398333 | 0.042874274 | novel_mir701 | 3.431349984 | 0.018074389 |
| novel_mir19 | 4.354934176 | 0.00357773 | novel_mir703 | 4.396527266 | 0.011670132 |
| novel_mir190 | 3.537558696 | 0.035993979 | novel_mir708 | 3.472707514 | 0.042209972 |
| novel_mir193 | 5.230776151 | 0.022358035 | novel_mir714 | 2.995905702 | 0.044231306 |
| novel_mir197 | 3.293531847 | 0.038824801 | novel_mir715 | 6.257334304 | 0.002420584 |
| novel_mir199 | 4.161235531 | 0.013094823 | novel_mir721 | 4.008318765 | 0.011297211 |
| novel_mir2 | 3.733652055 | 0.015178926 | novel_mir725 | 6.054228793 | 0.024044499 |
| novel_mir202 | 3.80914967 | 0.012566742 | novel_mir742 | 4.521325088 | 0.008028767 |
| novel_mir203 | 3.383888891 | 0.049017164 | novel_mir743 | 5.36270879 | 0.042912793 |
| novel_mir208 | 6.406158106 | 0.027715489 | novel_mir744 | 6.801780469 | 0.00852895 |
| novel_mir21 | 3.260137636 | 0.041777309 | novel_mir746 | 3.858741553 | 0.018760816 |
| novel_mir210 | 6.149637989 | 0.001141131 | novel_mir747 | 4.644287514 | 0.010816935 |
| novel_mir212 | 5.810342523 | 0.003104558 | novel_mir748 | 6.595233127 | 0.008008826 |
| novel_mir215 | 4.185566688 | 0.008850078 | novel_mir753 | 4.298061382 | 0.013389773 |
| novel_mir217 | 3.896852457 | 0.025313307 | novel_mir755 | 4.81531346 | 0.005868822 |
| novel_mir22 | 7.907308414 | 0.000958008 | novel_mir757 | 6.754362146 | 9.25E-05 |
| novel_mir223 | 6.16406 | 0.018863906 | novel_mir759 | 4.251798691 | 0.003602044 |
| novel_mir224 | 3.917900006 | 0.033236269 | novel_mir766 | 6.897873491 | 0.001922604 |
| novel_mir225 | 4.205814467 | 0.006958358 | novel_mir768 | -7.033254128 | 0.001534141 |
| novel_mir23 | 4.546144306 | 0.005579056 | novel_mir769 | 3.119735826 | 0.038186752 |
| novel_mir233 | 6.055905294 | 0.047693793 | novel_mir77 | -6.820046126 | 0.009721727 |
| novel_mir234 | 4.212070806 | 0.048069469 | novel_mir772 | 6.402146797 | 0.012026456 |
| novel_mir239 | 6.386370231 | 0.015943317 | novel_mir777 | 6.108586327 | 0.004899067 |
| novel_mir24 | 6.736160366 | 0.000850143 | novel_mir779 | 6.598010159 | 0.002251195 |
| novel_mir244 | 3.72662479 | 0.028400606 | novel_mir781 | 4.453553815 | 0.00849292 |
| novel_mir247 | 4.50751406 | 0.004788247 | novel_mir791 | 7.176124727 | 0.003394972 |
| novel_mir248 | 5.369907943 | 0.016910853 | novel_mir793 | 4.661962933 | 0.004949592 |
| novel_mir249 | 3.814650364 | 0.018221688 | novel_mir794 | 4.106810573 | 0.008060523 |
| novel_mir254 | 5.76924736 | 0.010449371 | novel_mir796 | 3.25207918 | 0.042148466 |
| novel_mir258 | 7.370684032 | 0.001979545 | novel_mir799 | 4.305488114 | 0.009197448 |
| novel_mir259 | 8.035201584 | 0.0014653 | novel_mir8 | 3.309628239 | 0.04833404 |
| novel_mir261 | 5.326132369 | 0.00337636 | novel_mir800 | 5.788906216 | 0.008056625 |
| novel_mir263 | 4.660016539 | 0.001794804 | novel_mir804 | 3.242411987 | 0.042376599 |
| novel_mir264 | 7.945076048 | 0.000737579 | novel_mir806 | 3.554992293 | 0.016526127 |
| novel_mir268 | 4.637901529 | 0.019685041 | novel_mir81 | 6.197018163 | 0.000157716 |
| novel_mir277 | 6.207279912 | 0.017609837 | novel_mir813 | 9.405433694 | 3.04E-07 |
| novel_mir279 | 5.027848519 | 0.039792969 | novel_mir816 | 4.543369336 | 0.028579339 |
| novel_mir28 | 3.353137125 | 0.02776394 | novel_mir822 | 5.75489353 | 0.000381623 |
| novel_mir283 | 7.234196402 | 0.00145638 | novel_mir824 | 4.632314865 | 0.000802004 |
| novel_mir290 | 7.412753616 | 0.004741471 | novel_mir833 | 3.225982288 | 0.034876441 |
| novel_mir291 | 6.142830227 | 0.022011887 | novel_mir839 | 2.623142774 | 0.049218874 |
| novel_mir294 | 5.598796205 | 0.001294619 | novel_mir844 | 8.309040317 | 0.000122717 |
| novel_mir296 | 4.696112778 | 0.016292986 | novel_mir846 | -8.216190325 | 0.000852424 |
| novel_mir299 | 3.233144158 | 0.019399666 | novel_mir847 | 9.095018689 | 8.70E-05 |
| novel_mir3 | 8.042831613 | 6.94E-06 | novel_mir851 | 5.09615684 | 0.000996024 |
| novel_mir301 | 5.396296207 | 0.000249478 | novel_mir856 | 4.191470486 | 0.009013396 |
| novel_mir309 | 4.82684645 | 0.012874967 | novel_mir857 | 4.802954064 | 0.0019298 |
| novel_mir310 | 6.648349417 | 0.006296516 | novel_mir864 | 3.353410982 | 0.045779913 |
| novel_mir311 | 6.066959583 | 0.002248178 | novel_mir866 | 3.919930892 | 0.002825591 |
| novel_mir314 | 6.013794808 | 0.005851258 | novel_mir87 | 6.932880544 | 0.000811455 |
| novel_mir317 | 4.375530728 | 0.026688244 | novel_mir872 | 4.868629425 | 0.002862358 |
| novel_mir318 | 3.420486855 | 0.021714829 | novel_mir873 | 6.230974232 | 0.016706839 |
| novel_mir319 | 4.619247813 | 0.029174271 | novel_mir879 | 6.907630021 | 0.000697009 |
| novel_mir320 | 4.557046405 | 0.012558081 | novel_mir88 | 3.46182434 | 0.026034062 |
| novel_mir324 | 5.227514294 | 0.003011879 | novel_mir880 | 7.135027409 | 0.00177287 |
| novel_mir325 | 4.058407614 | 0.021616431 | novel_mir882 | 7.048436 | 0.002312634 |
| novel_mir327 | 3.351256252 | 0.045350592 | novel_mir887 | 5.373435259 | 0.016933353 |
| novel_mir328 | 3.158432707 | 0.034439974 | novel_mir92 | 9.062885695 | 3.55E-05 |
| novel_mir33 | 6.698659924 | 0.000282848 | novel_mir95 | 7.993210391 | 0.000619456 |
| novel_mir330 | 5.591394325 | 0.000136713 | novel_mir96 | 5.483651174 | 0.021249081 |

**Table S3. immune cell infiltrations by xCell**

|  | Sham1.FPKM | Sham2.FPKM | CLP1.FPKM | CLP2.FPKM |
| --- | --- | --- | --- | --- |
| Adipocytes | 0 | 0 | 0 | 0 |
| Astrocytes | 0 | 0 | 0 | 0 |
| B-cells | 0.0088 | 0.005 | 0 | 0.0049 |
| Basophils | 0.0241 | 0.0198 | 0.001 | 0 |
| CD4+ T-cells | 0 | 0 | 0 | 9.00E-04 |
| CD4+ Tcm | 0 | 0 | 0 | 0 |
| CD4+ Tem | 0.0219 | 0.0196 | 0 | 0.0203 |
| CD4+ memory T-cells | 5.00E-04 | 0.004 | 0 | 0 |
| CD4+ naive T-cells | 0 | 0 | 0 | 0 |
| CD8+ T-cells | 0 | 0 | 0 | 9.00E-04 |
| CD8+ Tcm | 0 | 0 | 0 | 0 |
| CD8+ Tem | 0 | 0 | 0 | 0 |
| CD8+ naive T-cells | 0.036 | 0.0176 | 0 | 0.0162 |
| CLP | 0 | 0.0411 | 0.062 | 0.0378 |
| CMP | 0.0158 | 0.0197 | 0 | 0.0083 |
| Chondrocytes | 0 | 0.002 | 0 | 0.0099 |
| Class-switched memory B-cells | 0 | 0.0092 | 0 | 0 |
| DC | 8.00E-04 | 0 | 0.0157 | 0.016 |
| Endothelial cells | 0.0029 | 0 | 5.00E-04 | 0 |
| Eosinophils | 0 | 0 | 0 | 0 |
| Epithelial cells | 0.0016 | 0.0076 | 0.0019 | 2.00E-04 |
| Erythrocytes | 0 | 0 | 0 | 0 |
| Fibroblasts | 0 | 0.0426 | 0.0171 | 0.023 |
| GMP | 0 | 0.0087 | 0 | 0 |
| HSC | 0 | 0 | 0.4147 | 0.0762 |
| Hepatocytes | 0 | 0 | 0.0015 | 0 |
| Keratinocytes | 0 | 0 | 0 | 0 |
| MEP | 0.0615 | 0.0377 | 0.0831 | 0.0421 |
| MPP | 0.0128 | 0 | 0 | 0 |
| MSC | 0 | 0.0208 | 0 | 0.0506 |
| Macrophages | 0 | 0.0093 | 0.0039 | 0 |
| Macrophages M1 | 0 | 0.0042 | 0.0178 | 0.0028 |
| Macrophages M2 | 0.0143 | 0.0191 | 0.0141 | 0.0033 |
| Mast cells | 0.0037 | 0 | 0 | 0 |
| Megakaryocytes | 0 | 0 | 0 | 0 |
| Melanocytes | 6.00E-04 | 0.0037 | 0 | 4.00E-04 |
| Memory B-cells | 0 | 0 | 0 | 0 |
| Mesangial cells | 0.0049 | 0 | 0 | 0 |
| Monocytes | 0 | 0 | 0 | 0 |
| Myocytes | 0.0011 | 0.0108 | 0.0169 | 0.0031 |
| NK cells | 0 | 0 | 0 | 0 |
| NKT | 0.0375 | 0 | 0.0357 | 0 |
| Neurons | 0.0016 | 0 | 0.001 | 9.00E-04 |
| Neutrophils | 1.00E-04 | 0 | 0.0016 | 0 |
| Osteoblast | 0 | 0 | 0.0322 | 0.0034 |
| Pericytes | 0 | 0 | 0 | 0.039 |
| Plasma cells | 0.0156 | 0.0213 | 0.0076 | 0.0219 |
| Platelets | 0 | 8.00E-04 | 0 | 0 |
| Preadipocytes | 0.0242 | 0 | 0 | 0 |
| Sebocytes | 0 | 0 | 0 | 0 |
| Skeletal muscle | 0 | 0.0047 | 0.0053 | 0.0039 |
| Smooth muscle | 0.0604 | 0.0409 | 0.0732 | 0.0341 |
| Tgd cells | 0 | 0 | 0 | 0 |
| Th1 cells | 0.1238 | 0.1288 | 0.0809 | 0.0698 |
| Th2 cells | 0 | 0.0322 | 0.0661 | 0.0921 |
| Tregs | 0 | 0 | 0 | 0 |
| aDC | 0 | 0 | 0.2587 | 0.1297 |
| cDC | 0.0178 | 0.0045 | 0.0031 | 0 |
| iDC | 0.168 | 0.0487 | 0 | 0 |
| ly Endothelial cells | 0.0082 | 0.0087 | 0.0205 | 0 |
| mv Endothelial cells | 0.0159 | 0.0085 | 0.006 | 0.0092 |
| naive B-cells | 0 | 0 | 0 | 0 |
| pDC | 3.00E-04 | 0.0048 | 0 | 0 |
| pro B-cells | 0.0273 | 3.00E-04 | 0 | 0 |
| ImmuneScore | 0.0089 | 0.0095 | 0.0141 | 0.0151 |
| StromaScore | 0.0014 | 0.0213 | 0.0088 | 0.0115 |
| MicroenvironmentScore | 0.0104 | 0.0308 | 0.0229 | 0.0266 |

**Table S4. Upregulated and downregulated miRNAs in RAW264.7 cells (|Fold Change|>1.5, p>0.05)**

| miRNAs | logFC | P value | miRNAs | logFC | P value |
| --- | --- | --- | --- | --- | --- |
| novel-mmu-miR337-3p | -4.428666315 | 0.00123366 | mmu-miR-5627-5p | 2.596012174 | 0.013842183 |
| mmu-miR-214-3p | -3.855840688 | 0.00130096 | mmu-miR-1943-3p | 2.368773878 | 0.016359433 |
| mmu-miR-331-5p | 4.003347991 | 0.001794845 | novel-mmu-miR104-3p | -3.323714009 | 0.019155869 |
| mmu-miR-6539 | -3.716777852 | 0.002534148 | novel-mmu-miR177-3p | -2.392925008 | 0.020033605 |
| novel-mmu-miR312-5p | 3.612557982 | 0.002327065 | mmu-miR-92b-5p | -1.776790296 | 0.026632696 |
| novel-mmu-miR22-3p | 3.607461465 | 0.002423935 | mmu-miR-672-5p | 2.263317463 | 0.027938138 |
| mmu-miR-6418-3p | 3.501246828 | 0.00236763 | novel-mmu-miR478-5p | 2.61259872 | 0.028419523 |
| novel-mmu-miR384-5p | -3.866368683 | 0.003209009 | mmu-miR-6977-5p | 2.746517782 | 0.029663774 |
| novel-mmu-miR74-5p | 3.3939856 | 0.003086178 | mmu-miR-6993-5p | 4.511276949 | 0.031290394 |
| novel-mmu-miR183-5p | 3.327796918 | 0.004206644 | novel-mmu-miR9-5p | 2.00659981 | 0.032241066 |
| mmu-miR-1198-3p | 3.198195673 | 0.0042876 | mmu-miR-7034-3p | 2.00659981 | 0.032241066 |
| novel-mmu-miR432-3p | 3.198195673 | 0.0042876 | mmu-miR-7009-5p | 1.506445641 | 0.031981149 |
| mmu-miR-5119 | -2.381089639 | 0.006238733 | novel-mmu-miR193-5p | 1.886006616 | 0.032862548 |
| mmu-miR-6988-3p | 3.018910292 | 0.006380867 | mmu-miR-201-5p | -2.663364494 | 0.037220314 |
| mmu-miR-5122 | -3.489075439 | 0.007980255 | novel-mmu-miR252-3p | 2.631181106 | 0.037818806 |
| novel-mmu-miR302-3p | 2.850518419 | 0.007096572 | mmu-miR-6940-3p | -2.606414968 | 0.041316507 |
| novel-mmu-miR255-5p | 2.850518419 | 0.007096572 | mmu-miR-148a-3p | 2.222050022 | 0.041517808 |
| mmu-miR-3061 | -3.38547534 | 0.008356708 | novel-mmu-miR100-5p | 1.998893864 | 0.04148567 |
| mmu-miR-361-3p | -2.756413889 | 0.008170429 | novel-mmu-miR226-5p | 1.998893864 | 0.04148567 |
| novel-mmu-miR61-3p | 2.81478954 | 0.009123932 | novel-mmu-miR346-5p | -1.83877781 | 0.03843611 |
| mmu-miR-7043-3p | 3.516007908 | 0.010846115 | mmu-miR-6943-3p | -1.9074942 | 0.044654133 |
| mmu-miR-6395 | -3.664426965 | 0.011560046 | novel-mmu-miR16-5p | -1.901934967 | 0.045489237 |
| mmu-miR-7653-3p | 2.621737273 | 0.010701754 | mmu-miR-122-5p | 2.880942413 | 0.046265661 |
| mmu-miR-10a-3p | 2.621737273 | 0.010701754 | mmu-miR-199a-3p | 1.64140996 | 0.049774691 |
| novel-mmu-miR68-3p | 3.142512278 | 0.013778309 | mmu-miR-7054-3p | 1.64140996 | 0.049774691 |
| mmu-miR-5100 | 2.596012174 | 0.013842183 | novel-mmu-miR242-3p | 1.64140996 | 0.049774691 |
|  |  |  | mmu-miR-7667-5p | 1.791762425 | 0.047731572 |

**Table S5. Downregulated miRNAs** **expression matrix**

|  | Sham1 | Sham2 | CLP1 | CLP2 | CLP3 |
| --- | --- | --- | --- | --- | --- |
| miRNA id | cout | cout | cout | cout | cout |
| mmu-miR-7115-5p | 3 | 1 |  | 2 | 1 |
| mmu-miR-194-5p | 1690001 | 488392 | 124430 | 964773 | 200146 |
| mmu-miR-5619-3p | 9 | 8 | 1 | 1 |  |
| mmu-miR-3964 | 14 | 12 | 2 | 3 | 5 |
| mmu-miR-5114 | 17 | 14 | 1 | 11 | 3 |
| mmu-let-7j | 11815 | 616 | 350 | 16855 | 11780 |
| mmu-miR-192-5p | 1655086 | 1399051 | 462443 | 199828 | 475511 |
| mmu-miR-669p-5p | 123 | 164 | 11 | 31 | 37 |
| mmu-let-7b-5p | 194382 | 135497 | 19301 | 23817 | 16464 |
| mmu-miR-7213-3p | 9 | 5 | 4 | 9 | 3 |
| mmu-miR-3061 | 63 | 29 | 18 | 55 | 32 |
| mmu-miR-6934-3p | 7 | 1 | 3 | 1 | 4 |
| mmu-miR-7649-3p | 7 | 12 | 21 | 9 | 1 |
| mmu-miR-205-5p | 201 | 20 | 21 | 30 | 75 |
| mmu-miR-186-5p | 379 | 457 | 136 | 416 | 313 |
| mmu-miR-877-5p | 214 | 67 | 52 | 57 | 36 |
| mmu-miR-5107-3p | 21 | 32 | 2 | 21 | 17 |
| mmu-miR-146a-5p | 19116 | 24653 | 4768 | 14989 | 19668 |
| mmu-miR-7115-3p | 5 | 1 | 2 | 3 | 2 |
| mmu-miR-5134-3p | 5 | 13 | 4 | 4 | 5 |
| mmu-miR-467d-5p | 269 | 676 | 22 | 198 | 92 |
| mmu-miR-27a-5p | 237 | 37 | 229 | 361 | 48 |
| mmu-miR-7032-3p | 9 | 18 | 3 | 6 | 4 |
| mmu-miR-505-3p | 113 | 53 | 19 | 91 | 29 |
| mmu-miR-466c-5p | 112 | 213 | 9 | 126 | 57 |
| mmu-let-7d-5p | 134936 | 135307 | 24734 | 64507 | 62099 |
| mmu-miR-141-5p | 1637 | 2728 | 741 | 2158 | 2228 |
| mmu-miR-7051-5p | 9 | 1 | 1 | 3 | 5 |
| mmu-miR-200b-5p | 3910 | 4115 | 1925 | 5781 | 4351 |
| mmu-miR-1224-3p | 28 | 8 | 3 | 31 | 14 |
| mmu-miR-15b-5p | 28967 | 30971 | 1542 | 25588 | 20505 |
| mmu-miR-7213-5p | 2 | 8 | 2 | 5 | 6 |
| mmu-miR-5134-5p | 4 | 2 | 2 | 5 | 1 |
| mmu-miR-184-3p | 180 | 88 | 43 | 156 | 69 |
| mmu-miR-6971-3p | 5 | 9 | 1 | 3 | 2 |
| mmu-miR-1981-5p | 7300 | 713 | 907 | 3212 | 1920 |
| mmu-miR-181b-2-3p | 13467 | 8908 | 4795 | 12429 | 12242 |
| mmu-miR-93-5p | 71073 | 41417 | 7094 | 52972 | 33781 |
| mmu-miR-129b-3p | 31 | 548 | 72 | 929 | 870 |
| mmu-miR-1934-5p | 14 | 15 | 5 | 17 | 15 |
| mmu-miR-466h-5p | 22 | 44 | 6 | 44 | 12 |
| mmu-miR-218-5p | 8059 | 895 | 587 | 7414 | 5965 |
| mmu-miR-3066-5p | 21 | 21 | 13 | 1 | 17 |
| mmu-miR-1948-3p | 116 | 50 | 23 | 53 | 20 |

**Table S5. qPCR Primers for mRNAs (mouse)**

| **Target genes** | **Forward primers (5′‐3′)** | **Reverse primers (3′‐5′)** |
| --- | --- | --- |
| TNF-α | AAGGCCGGGGTGTCCTGGAG | AGGCCAGGTGGGGACAGCTC |
| IL-1β | GAAATGCCACCTTTTGACAGTG | TGGATGCTCTCATCAGGACAG |
| IL-6 | GACTGATGCTGGTGACAACC | GACAGGTCTGTTGGGAGTG |
| iNOS | TTTGACGCTCGGAACTGTAG | GAAGTCATGTTTGCCGTCAC |
| CCR7 | GGTGGCTCTCCTTGTCATTTTC | AGGTTGAGCAGGTAGGTATCCG |
| Arg1 | ACCTGGCCTTTGTTGATGTC | CAGCACCACACTGACTCTTC |
| Fizz1 | CCCTTCTCATCTGCATCTCC | CAGTAGCAGTCATCCCAGCA |
| Snail1 | CACACGCTGCCTTGTGTCT | GGTCAGCAAAAGCACGGTT |
| β-Actin | GTGACGTTGACATCCGTAAAGA | GTAACAGTCCGCCTAGAAGCAC |
| U6 | ATTGGAACGATACAGAGAAGATT | GGAACGCTTCACGAATTTG |

**Table S6. qPCR Primers for miRNAs**

| **Target genes** | **Accession no.** | **Sequence (5′‐3′)** |
| --- | --- | --- |
| miR-3061-3p | MIMAT0014829 | CAGCTACCTTTGATAGTCCAC |
| miR-505-3p | MIMAT0003513 | CGTCAACACTTGCTGGT |
| miR-5114 | MIMAT0020622 | ACTGGAGACGGAAGCTG |
| miR-184-3p | MIMAT0000213 | TGGACGGAGAACTGATAAGGGT |
| miR-27a-5p | MIMAT0004633 | AGGGCTTAGCTGCTTGT |
| miR-15b-5p | MIMAT0000124 | GCAGTAGCAGCACATCA |
| miR-5107-5p | MIMAT0020615 | GGGCAGAGGAGGCA |

**Supplemental Figures**

**
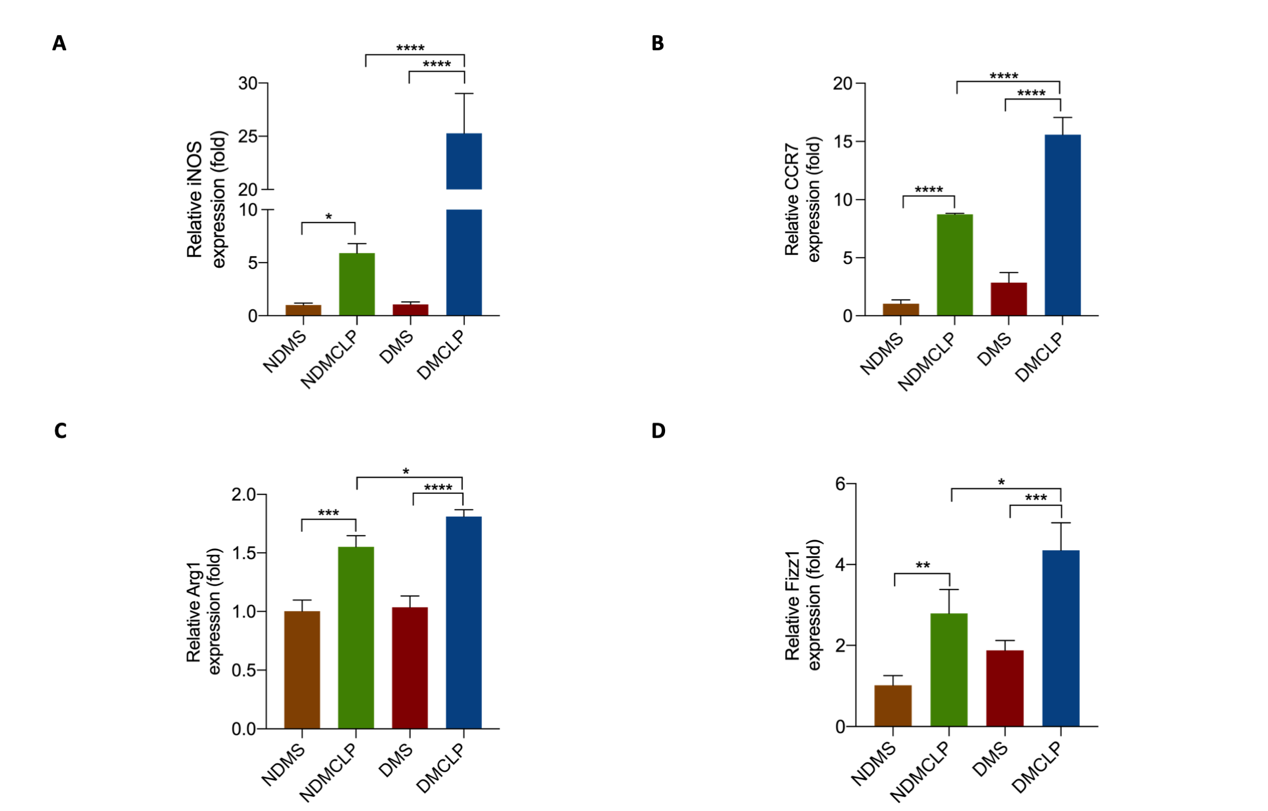
**

**Figure S1. The mRNA expression of M1 and M2 macrophage markers in intestinal tissue. (related to Figure 1)**

A and B. The M1 markers iNOS and CRR7 were measured by qRT-PCR. C and D. The M1 markers Arg1 and Fizz1 were measured by qRT-PCR. *p < 0.05, **p < 0.001, ***p < 0.001, and ****p < 0.0001.


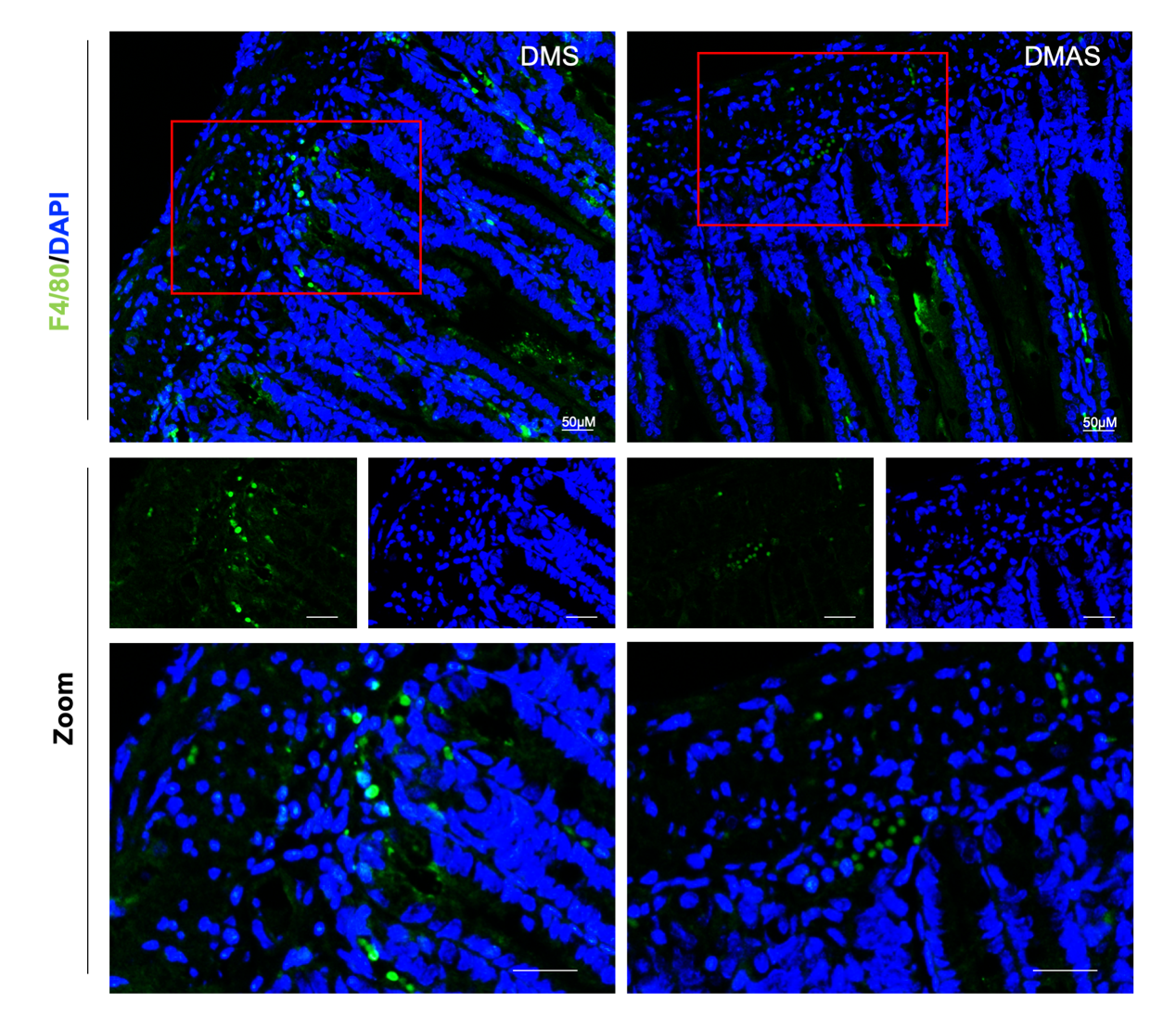


**Figure S2. Immunofluorescence staining of F4/80 (Green) in intestinal issues of DMS and DMAS groups. (related to Figure 2)**


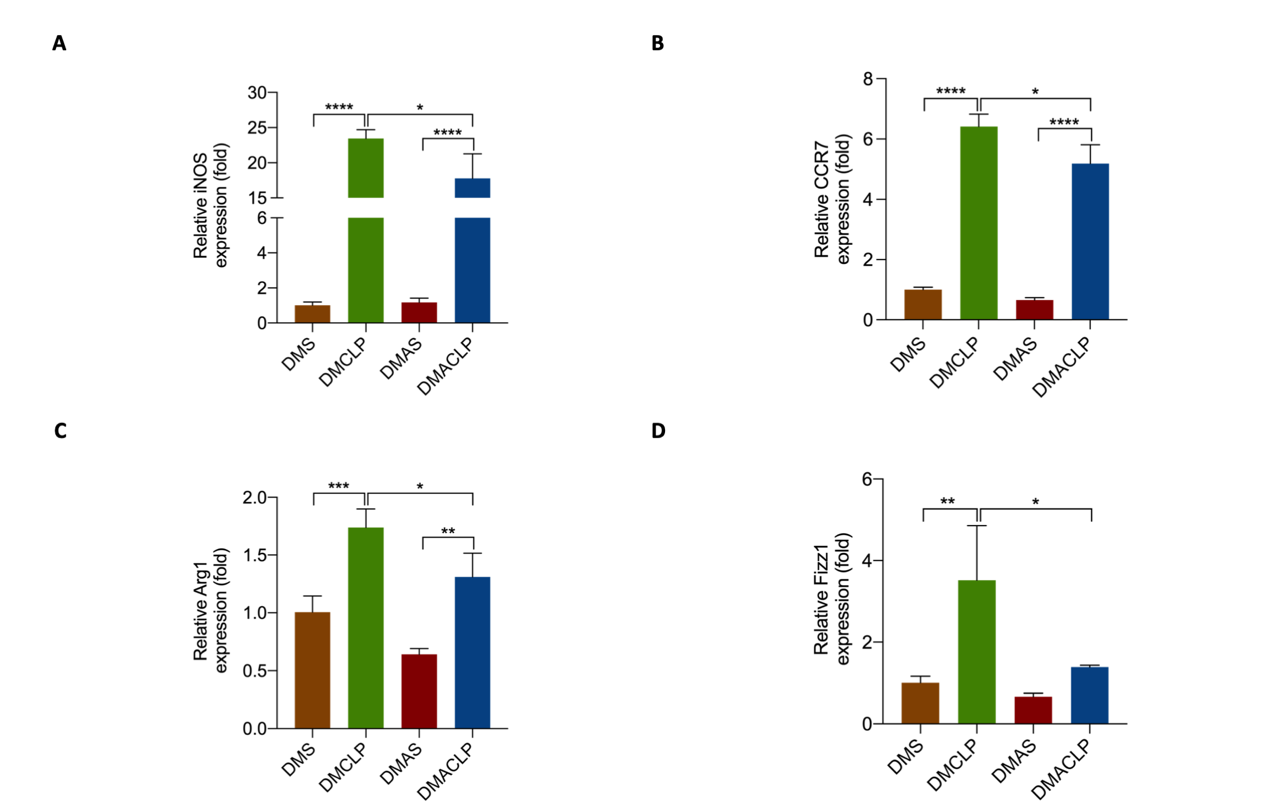


**Figure S3. The mRNA expression of M1 and M2 macrophage markers in intestinal tissue after macrophages depletion. (related to Figure 2)**

A and B. The M1 markers iNOS and CCR7 were measured by qRT-PCR. C and D. The M1 markers Arg1 and Fizz1 were measured by qRT-PCR. *p < 0.05, **p < 0.001, ***p < 0.001, and ****p < 0.0001.


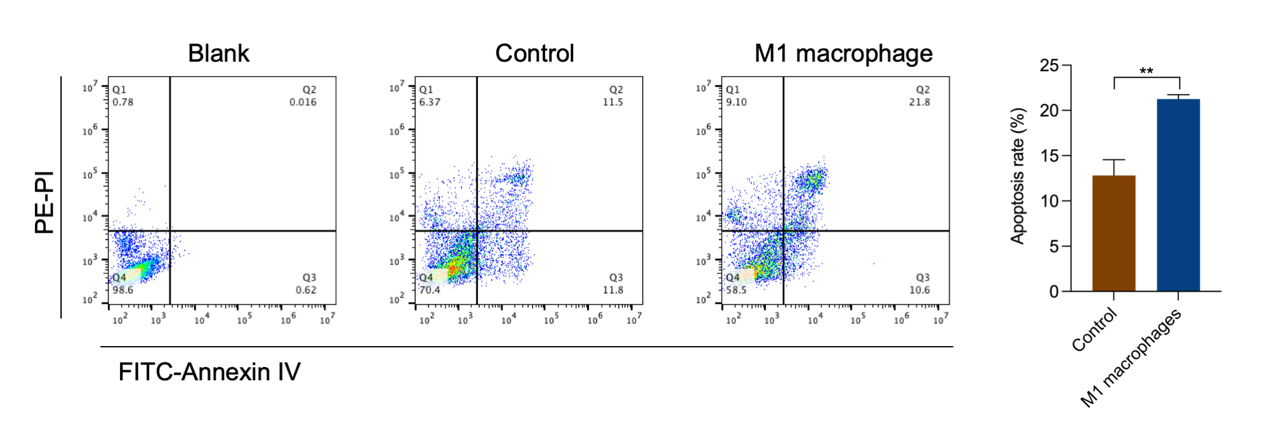


**Figure S4. LPS-stimulated M1 macrophages increased apoptosis of intestinal mucosal epithelial cells**

**
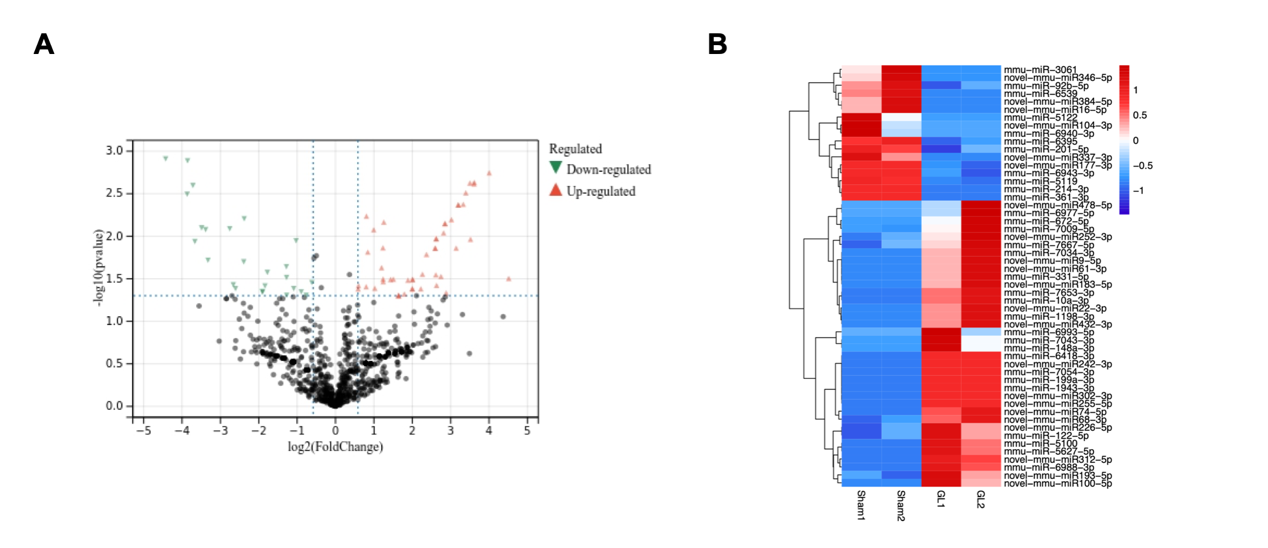
**

**Figure S5. miRNAs expression in HG+LPS stimulated RAW264.7 cells. (related to Figure 3)**

A. The volcano plot of miRNAs. The red dots illustrate upregulated miRNAs and the green dots display downregulated miRNAs. B Heat map of miRNA-seq. The fluorescence intensity of differentially expressed miRNAs (≥ 1.5 fold) is illustrated from high (red) to low (blue)..

**Figure S6.** **mRNAs expression**


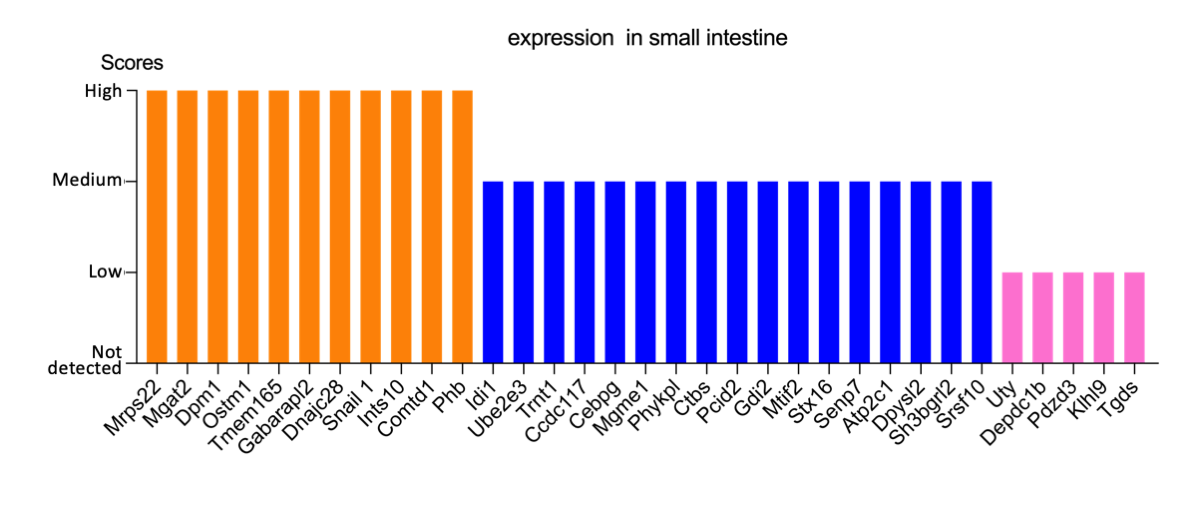


**Figure S7. mRNAs expression levels in small intestine in Proteinatlas database. (related to Figure 5)**


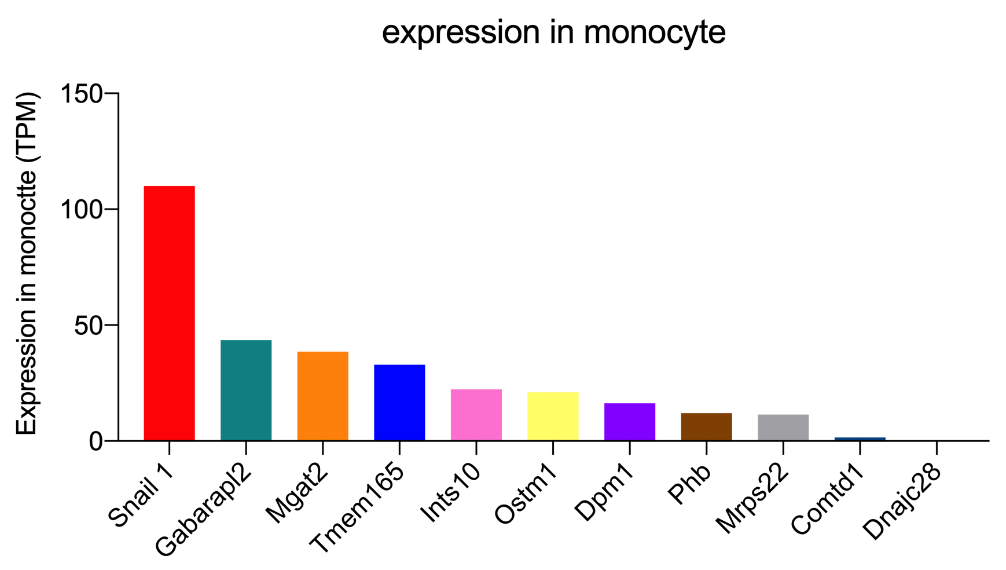


**Figure S8. mRNAs expression levels in monocyte cells in Proteinatlas database. (related to Figure 5)**


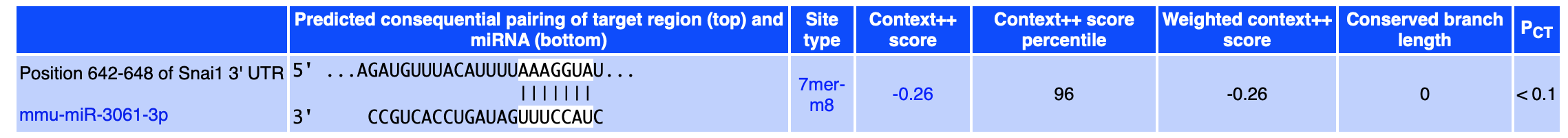


**Figure S9. The predicting binding site information between miR-3061 and Snail1 mRNA** **3’-UTR in TargetScan and miRDBdatabase. (related to Figure 5)**

**
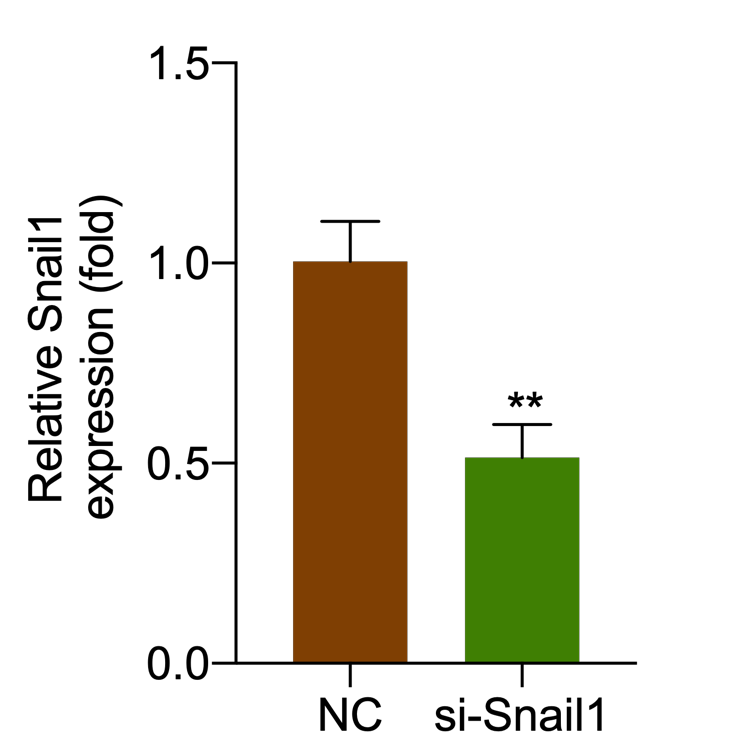
**

**Figure S10. The Snail1 expression after Snail1 siRNA transfection. (related to Figure 6)**

Snail1 mRNA expression was obviously decreased in the si-Snail1 group compared with NC group.

**Reference**

1. Moss M, Guidot DM, Steinberg KP, Duhon GF*, et al.* Diabetic patients have a decreased incidence of acute respiratory distress syndrome. Critical care medicine*.* 2000;28(7):2187-2192.

2. Jakubowska I, Lukasiewicz D. [Serious course of sepsis in diabetic patients]. Przeglad epidemiologiczny*.* 2006;60 Suppl 1:46-50.

3. Kofteridis DP, Papadimitraki E, Mantadakis E, Maraki S*, et al.* Effect of diabetes mellitus on the clinical and microbiological features of hospitalized elderly patients with acute pyelonephritis. Journal of the American Geriatrics Society*.* 2009;57(11):2125-2128.

4. van Vught LA, Wiewel MA, Klein Klouwenberg PM, Hoogendijk AJ*, et al.* Admission Hyperglycemia in Critically Ill Sepsis Patients: Association With Outcome and Host Response. Critical care medicine*.* 2016;44(7):1338-1346.

5. van Vught LA, Holman R, de Jonge E, de Keizer NF*, et al.* Diabetes Is Not Associated With Increased 90-Day Mortality Risk in Critically Ill Patients With Sepsis. Critical care medicine*.* 2017;45(10):e1026-e1035.

6. Zohar Y, Zilberman Itskovich S, Koren S, Zaidenstein R*, et al.* The association of diabetes and hyperglycemia with sepsis outcomes: a population-based cohort analysis. Internal and emergency medicine*.* 2021;16(3):719-728.
